# A crew-operated data recording system for length-based stock assessment of Indonesia’s deep demersal fisheries

**DOI:** 10.1371/journal.pone.0263646

**Published:** 2022-02-25

**Authors:** Elle Wibisono, Peter Mous, Edwison Firmana, Austin Humphries

**Affiliations:** 1 Department of Fisheries, Animal and Veterinary Sciences, University of Rhode Island, Kingston, Rhode Island, United States of America; 2 Yayasan Konservasi Alam Nusantara (YKAN) Fisheries Program, Bali, Indonesia; 3 Indonesia Ministry of Marine Affairs and Fisheries, Directorate General of Capture Fisheries, Jakarta, Indonesia; 4 Graduate School of Oceanography, University of Rhode Island, Narragansett, Rhode Island, United States of America; Aristotle University of Thessaloniki, GREECE

## Abstract

Deep demersal fisheries in Indonesia yielded close to 90,000 metric tons of snapper and grouper in 2019, landed by a fleet of approximately 10,000 fishing boats. Prior to the present study, information on these multi-species, dispersed, small- to medium-scale fisheries was scarce, while reliable species-specific data on catch and effort were non-existent. This data-deficiency made stock assessments and design of harvest control rules impossible. We developed a new data collection method, the Crew Operated Data Recording System (CODRS), to collect verifiable species- and length-composition data from catches across all segments of the fleet. CODRS engaged crews of 579 fishing vessels to take pictures of each fish in their catch, in combination with the deployment of a tracking device on their boats. Furthermore, we also conducted a frame survey to map the fleet across the entire Indonesian archipelago. Using more than 2 million CODRS images, we aimed to understand the basic characteristics and challenges within the fishery. We updated life-history parameters for the top 50 species in the fishery based on the maximum observed length-frequency distribution of the catch (i.e., asymptotic length, size at maturity, optimum fishing length, total mortality, and spawning potential ratio). Length-based stock assessments using the updated life-history parameters showed high risks of overfishing for most of the major target species, especially for snapper species with large maximum sizes. Our results indicated that effective management and harvest strategies are urgently needed across Indonesia’s eleven Fishery Management Areas to prevent the collapse of these important fisheries.

## Introduction

The deep demersal fisheries in Indonesia are of international and local importance for livelihood, economic output, and food security. They target snappers, groupers, grunts, emperors, croakers, and over 100 co-occurring species at depths ranging between 30 and 350 meters [1]. The fisheries are also multi-gear, with droplines and bottom-set longlines as the most common, followed by traps and gillnets. In addition, some fishers use droplines and longlines or traps and droplines concurrently. To ensure the sustainability of these disperse multi-species and multi-gear fisheries, it is important to have reliable data. To date, however, these fisheries are data-poor, as there are no accurate catch or effort statistics, population dynamics of target species are mostly unknown, and vessel dynamics remain elusive. Thus, a basic characterization of the fisheries are still lacking.

Data collection and monitoring of these fisheries are challenging for many reasons. First, fishing locations are spread out over the entire Indonesian Archipelago, which comprises parts of two FAO Major Fishing Areas (57—Eastern Indian Ocean, and 71 –the Western and Central Pacific Ocean), further subdivided by the Indonesia Ministry of Marine Affairs and Fisheries into eleven Fisheries Management Areas (FMA). For stock assessments, these fisheries are included in the commodity "ikan demersal" (demersal fish), which also includes a wide range of other bottom-dwelling fish species [2]. Second, Indonesia’s seas and oceans feature some of the most biodiverse marine ecosystems on earth, and this biodiversity is reflected in the species diversity of landings: nearly 3,000 species have been recorded from Indonesia’s fish markets [3]. Hence, species identification presents a special challenge for enumerators and observers. Published accuracy rates for observers vary between 98% for groundfish in the North Pacific and 80% for scientific observers working on a shark fishery in northern Australia [1,4]. We believe that accuracy rates in Indonesia’s fisheries are much lower, due to the high species diversity, lack of trained personnel, and ambiguous naming of fish species in Bahasa Indonesia. *Kakap Merah* (red snapper), for example, is used for at least six snappers of the genera *Etelis* and *Lutjanus* [3]. Even in the scientific literature, misidentifications are common. For instance, *Lutjanus malabaricus* is often misidentified as *L*. *sanguineus* (a species of the western Indian Ocean) among scientists as well as fish exporters [5].

In addition to the logistical constraints of data collection in these fisheries, most of the vessels are small (1–10 Gross Ton; GT) to medium (11–30 GT) scale, and they are dispersed over vast and remote stretches of coastline. In such situations, conventional catch- and effort-based methods suffer from problems with species and gear identification, limited access to landing sites, difficulties with defining units of effort, and lack of resources for the implementation of monitoring programs by qualified enumerators [6,7]. Accurate port sampling requires well-trained enumerators to be present at the site and time of landing, often at odd hours [8]. Since small-scale boats often land their catch in a very dispersed manner, outside of the main ports, sufficient catch enumeration is almost impossible. Furthermore, for boats making longer fishing trips, it is difficult to determine actual fishing locations at the time of landing unless the boat has a tracking system on board and the enumerator can access the data. Logbooks are unsuitable for small- to medium-scale fisheries since the logbook forms do not align well with fishing practices which are highly dispersed with no central location where fish are landed and recorded. Hence, in Indonesia, logbooks are often completed onshore by agents who take care of the paperwork (e.g., location of fishing grounds, port of origin, number of crew) for the fishing boats at ports [9]. Observer programs can only be implemented on larger vessels, which represent a minor part of Indonesia’s demersal fleet, require technical expertise, and implementation can be unsafe due to poor working conditions [10]. In Indonesia, the standard catch and effort monitoring system, which was designed in the mid-1970s, has not been successful in capturing data with sufficient resolution for accurate stock assessment in the small- to medium-scale deep demersal fisheries [8,11,12].

Advances in digital imaging have reduced limitations in the implementation of conventional fishery-dependent data collection methods. For example, to complement or replace observers on vessels, electronic monitoring (EM) systems utilize a photo or video recording device, GPS tracker, and sensors attached to fishing gear to record catches [7]. Depending on the size and gear type of the fishing vessel, EM systems can be implemented in different ways—from installing a video camera, to manually taking photographs of catches using a handheld camera, or using sensors that record position, speed, or even hydraulic pressure to monitor gear activity [13,14]. Scientists have also used vessel movement patterns from vessel monitoring systems (VMS) to determine fishing locations [15]. However, especially for mixed and multi-species fisheries, challenges in the accuracy and implementation of these systems still remain [16]. Electronic monitoring systems have not yet demonstrated their feasibility in complex multi-species tropical fisheries that span different gear types and vessel sizes. Especially for small fishing vessels, a complex and expensive system would have limited applicability.

To address the need for reliable data in the deep demersal fisheries of Indonesia, we developed a data recording system for species and length composition of commercial catches that is based on photographic records of the fish in the catch, resulting in verifiable data. This system, referred to as the Crew-Operated Data Recording System (CODRS), combines simple hand-operated cameras with GPS trackers to simultaneously record catch, time, and location. CODRS is an iteration of EM that is tailored to a multi-gear and multi-species fishery. Thus, the goal of this research is to estimate characteristics of the deep demersal fisheries using the best-possible data as a foundation of more robust studies and policy analysis in the future. Here, we (a) conducted a frame survey to estimate the total size of this fishery, (b) reviewed CODRS implementation and compared the accuracy of CODRS against ledger receipts (catch volumes from traders) to see how it differs from a more traditional fishery-dependent data collection methodology, and (c) report stock assessment findings from CODRS. The CODRS dataset included 2,881,519 individual length observations of fishes, which allowed us to set reliable life-history parameters for the top 50 species based on verifiable estimations of L_max_ with large sample sizes. We used CODRS data to characterize the fishery (Catch per Unit Effort (CPUE) and total catch) and conduct preliminary length-based stock assessments by analyzing the risk of overfishing of the top species using Spawning Potential Ratio (SPR) and trade limits.

## Methods

### Frame survey

The research was done in partnership with The Nature Conservancy (TNC), who implemented the Crew-Operated Data Recording System (CODRS). TNC has a Memorandum of Understanding (MOU) with the Indonesian Ministry of Marine Affairs and Fisheries and has completed all the paperwork to allow its operation in the country. Prior to deployment of CODRS at each site, TNC research analysts obtained permission from local authorities to proceed with the data collection system. In April 2020, TNC handed over the CODRS monitoring program to its affiliate Yayasan Konservasi Alam Nusantara (YKAN). A MOU between YKAN and the Indonesian Ministry of Marine Affairs and Fisheries is still in the process of becoming official, but was signed by all parties in August 2020. In all interactions with fishers and/or other stakeholders (e.g., fish processing plant employees, local government officials), the research team obtained oral consent from individuals to participate.

A frame survey is a census-based approach in which data is collected on all fishing gears and vessels that are operating within a pre-defined area of interest [17]. In Indonesia, the areas of interest are the 11 Fishery Management Areas (FMA, *Wilayah Pengelolaan Perikanan*), of which there are eight in FAO area 71 (Western Central Pacific Ocean) and three in FAO area 57 (Eastern Indian Ocean). Indonesia’s system of FMAs are the geographic basis for stock assessments and fisheries management, and therefore we used this system for structuring our survey design. Unfortunately, there are no official data on the number of boats that participate in deep demersal fisheries. Indonesia does have a system to record fishing activity, but this system cannot be used as a basis for a frame survey since it does not differentiate between fisheries. The system functions by passing on aggregated data from one administrative level to the next, which means that granularity on boats and gears is lost at higher administrative levels. While implementing the CODRS program, we conducted a frame survey covering the entire coastline of all major Indonesian islands and all 11 FMAs.

We implemented the frame survey by systematically expanding our network of stakeholders through interviews with fishers, fish processing companies, and government officials. We used our initial partner fishers and fish processing companies as a source of information on other fishers and companies from other areas that operate in the deep demersal fisheries. We were not able to standardize the interviewees because each fish processing company and governmental agency has different structures and willingness to talk to ’outsiders’. For example, some company owners were eager to share information but other owners were reluctant and instead let their purchasing manager communicate with the research technician.

We used the following methods to list sites where presence of deep demersal fisheries were likely. First, we asked the processing companies that produce snapper and grouper fillets and their suppliers where they obtained their fish. Second, we asked fishing communities that were involved in deep demersal fisheries or part of the CODRS program whether they knew of any other important harbors or landing sites. A team of 30 field technicians spread across the archipelago then followed up the leads with site visits to corroborate the presence of another company or fishing community targeting the deep demersal species. For each confirmed case, two senior technicians then visited the area to ensure assessment accuracy.

For each area, we also asked the opinion of government researchers (especially the Institute for Marine Fisheries Research, *Balai Riset Perikanan Laut*, BRPL), fishing harbor authorities (especially the Fisheries Surveillance Agency, *Pengawasan Sumber Daya Kelautan dan Perikanan*, PSDKP), and the agency responsible for issuance of catch certificates (the Fish Quarantine and Inspection Agency, *Badan Karantina Ikan*, *Pengendalian Mutu dan Keamanan Hasil Perikanan*). We asked fishing port authorities on the importance or size of the deep demersal fisheries in their jurisdiction. We also asked the Fish Quarantine and Inspection Agency for a list of companies in the deep demersal fisheries. The field technicians then called and visited each company to determine if the company participates in the fisheries and the scale of the operation. Finally, we used Google Earth (accessed 12 July 2019) to scan satellite imagery of the Indonesia coastline to find concentrations of fishing vessels, after which we used a combination of online research and field visits to ground-truth whether the vessels were engaged in the deep demersal fisheries. Though unconventional, this continuous effort since 2015 to aggregate data on the scale and spread of the fishery, have covered most of Indonesian ports and coastal communities. During the frame survey, data were collected on boat size, gear type, the port of registration, licenses for specific FMAs, captain contacts, and other details, for all fishing boats in the fleet. We also recorded whether boats participated during part of the year in other fisheries ("seasonal"), or whether they fished for deep demersal fishes only ("dedicated"). For example, the fishers from Galesong (South Sulawesi) target deep demersal fishes for part of the year, and for the other part, they switch to collecting eggs of flying fish.

Following practices by fisheries managers in Indonesia, we distinguished four boat size categories, including nano (<5 GT), small (5 - <10 GT), medium (10–30 GT), and large (>30 GT). We also distinguished four gear types: vertical drop lines, bottom-set longlines, deep-water gillnets, and traps. A fifth category of gear classification was needed to record operations using "mixed gear" when two or more of the gear types were used on the same trip and catches were not separated. To characterize the fisheries, we analyzed differences in catches per fleet segment (the combination of size category and fishing gear).

This frame survey included information for each fishing boat in the fleet regarding boat size, gear type, the port of registration, and main fishing locations. Origins of vessels were not always overlapping with their fishing locations. Information on the main fishing location for individual vessels was updated when vessels moved to other fishing locations.

### Catch composition survey

**Fig 1:**
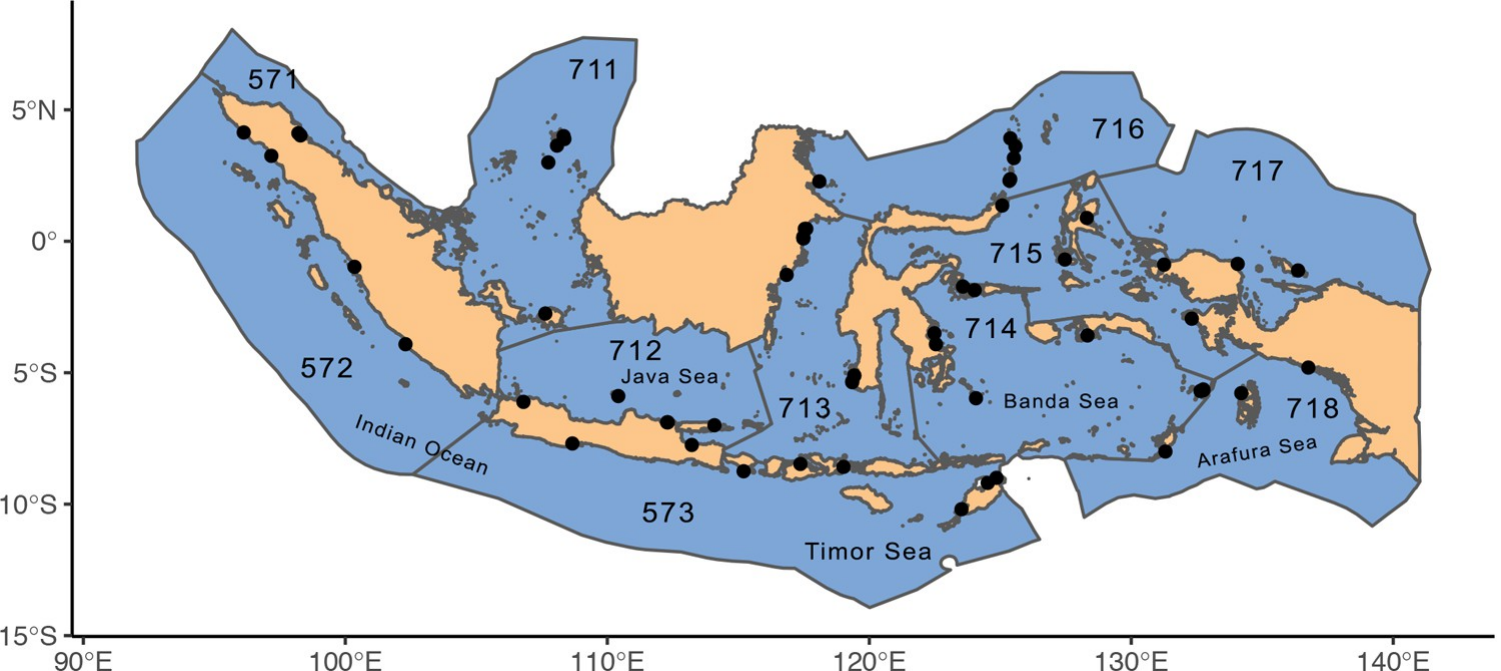
Map of the eleven Fishery Management Areas (FMA) within Indonesia. Black lines denote FMA boundaries. Dots denote fishing villages or ports where we deployed the Crew Operated Data Recording System (CODRS).

Between 2015 and March 2020, we implemented a catch composition monitoring program using CODRS. We started in FMA 573, and by the end of 2019, we had expanded the CODRS program to cover all of Indonesia’s eleven FMAs (Fig 1). The bathymetry of FMAs 573, 713, 714, 715, 716, and 717 is characterized by mostly narrow coastal shelves, seamounts, and deep trenches. The bathymetry of FMAs 711, 712, and 718 is mostly comprised of shallow waters over continental shelves (30 to 100 m depth). FMAs 571 and 572 have a mix of shallower continental shelf habitat and deeper slopes and drop-offs in the Indian Ocean and Malacca Strait, around the island of Sumatra.

The CODRS method is comparable to a logbook because it is based on data collection by fishers. We aimed to work with about 5% of the fleet operating on deep demersal fisheries, which amounts to about 40 vessels per FMA. We selected fishers based on their representativeness for the gears and boat size categories that operate in that FMA, with at least one, and where possible, multiple CODRS vessels within the same fleet segment. Since data from the frame survey became available during implementation of CODRS, we could not always ensure that selection of CODRS was completely representative for the fleet operating in the FMA, and we frequently had to make adjustments by focusing on recruiting under-represented fleets based on new insights from the frame survey.

As an incentive for collaboration, we provided captains with monthly compensation, scaled to their vessel size. As part of the selection process, we informed fishers of the data confidentiality—only pooled data are shared with other parties, and locations are only accessible by family members and boat owners. To ensure data quality, we terminated the contract with captains that violated the best practices (i.e., did not take complete photographs of the catch, took blurry or angled photographs).

**Fig 2:**
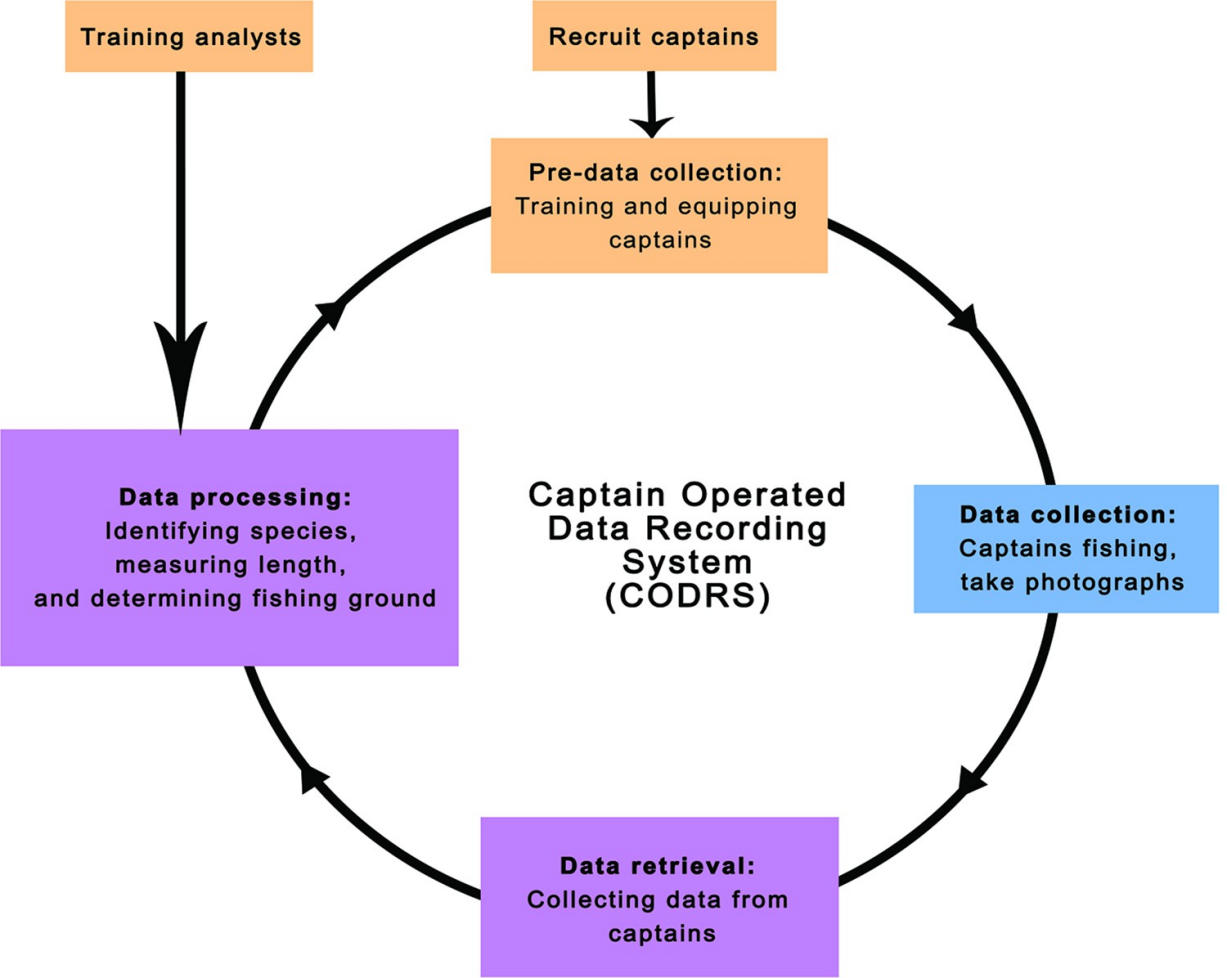
Crew-Operated Data Recording System (CODRS) workflow. The system is a cycle that begins with recruitment and training of captains and analysts (orange boxes). Data is then collected at sea (blue box), and transferred to analysts for processing (purple boxes).

We equipped CODRS vessels with digital pocket cameras and a measuring board for size reference, and we asked the fishers to take a picture of every fish they caught. With the exception of the crew of nano vessels, who often took pictures upon landing, crew of all other fishing vessels took pictures while at sea and were instructed to take pictures where they caught the fish. However, the pictures were often taken when transferring the catch in the hold. The fishers placed each fish on a measuring board for size reference. The acrylic measuring board had 1-cm grid lines across the width of the board; we color-coded every 10 cm mark to aid in data analysis of photographs. We installed a backstop at the 0-cm mark where the mouth of the fish should touch to decrease measurement error. In addition, we deployed a low-cost GPS tracking device (Spot Trace) to record positions, and we trained the captains to change batteries and take care of these devices. We set the devices to record a position every hour when moving. Spot Trace could record higher frequencies for recording of positions, but this feature reduced the battery life too much. We trained and assigned one technician per 15 vessels participating in the program. The technicians maintained relationships with captains and crew, and they received the digital media with the pictures from the captains after each trip. Research technicians provided feedback on the data quality to the captains after each fishing trip, comparing the number of pictures with a rough estimate of the volume of the catch. We also trained technicians in fish identification using published guides, frozen specimens, and photographs, so they could identify fish species, after which they measured length and input the data into spreadsheets (Fig 2).

Data collection for each trip began when the boat leaves port with the GPS recording vessel location every hour while it is steaming out. At the end of each fishing trip, which varied between a single day and two months depending on vessel size, captains transfer the memory card containing the photographs of their catch to the technicians onshore. Technicians then identified the species of each fish in the images and determined each specimen’s total length (TL; cm). After the first round of image processing by a field technician, experienced senior technicians reviewed the species identification and length measurement data for accuracy before adding it to the database. For any images of specimens exceeding the previous largest fish of that species in the database, a senior fisheries scientist further verified the image before accepting it as a new estimate for maximum length (L_max_). Based on the quality of the photographs, technicians also provided feedback to the fishers to improve data quality on subsequent trips. Sets of images from fishing trips with unacceptable low-quality photographs or only representing a small part of a multi-day fishing trip were not processed and not included in the dataset.

Technicians used sales ledgers (receipts) of the landing for quality control of CODRS data, assessing whether the CODRS images accounted for the entire catch. Technicians flagged catches when they were deemed incomplete. Datasets with photographs that represented >90% of catch receipts, 30% to 90% of catch receipts, and <30% of catch receipt were flagged as complete, incomplete, and biased, respectively. Only complete datasets were used for CPUE calculation and analyses, however, complete and incomplete datasets were used for the length-based stock assessment. Biased datasets were rejected and were not used for analysis.

After a dataset passed all reviews, and any necessary corrections were made, the dataset was uploaded to an online database. Vessel owners, captains, and researchers have access to the contents of the database, each with different viewing privileges. For instance, captains were not able to see the fishing locations and corresponding catches of other captains, but researchers were able to access all information except identifying characteristics such as boat/captain name. Fish traders who own the vessels or fronted the operational fishing costs could be given access to selected information on their fleet.

To validate CODRS data in more detail, we compared CODRS data with catch weights from the sales ledgers (receipts). We collected receipts from fish traders that purchased fish from CODRS vessels between August and November 2017. These sales receipts were assumed to represent a reliable estimate of the total weight of an individual catch (from a single trip, and including all species) that is independent of CODRS data [18]. We compared these data to catch estimates (per trip) from the CODRS system using paired t-tests and linear regression. Data were inspected for normality and homogeneity of variance using a Shapiro-Wilks test.

### Fishery characteristics

To determine the fish body weight (kg) from TL data, allometric length-weight relationships were obtained from the literature to convert fish sizes taken from the CODRS images (S1 Table). When no values were found for a species, we used morphologically similar species to obtain the length-weight coefficients. To determine fishing locations, we filtered GPS data based on speed (<5 km/h) and depth (50-500m). We assumed that boats traveling less than 5 km/h were always fishing, which may not always be the case, thus potentially causing a slight overestimation. Boats that might be fishing while drifting with the engines off would still fall into this category as long as their drift speed was less than 5 km/h.

To estimate the yearly number of fishing days, we categorized each day for which a CODRS vessel took a picture as a "fishing day", and we counted the number of fishing days for each CODRS vessel. We then calculated average fishing days in a year for each fleet segment (the combination of size category and fishing gear) (S2 Table). For each fleet segment, we assumed that "seasonal" fishers allocate half of the fishing days of "dedicated" fishers to deep-water demersal fisheries, and the other half to the other fisheries they participate in. Dedicated fishing boats on average were fishing actively between 200 and 250 days per year.

### Using Catch per Unit Effort (CPUE) to estimate total catch

We calculated the CPUE in order to estimate the total catch of the entire fleet of the fishery. We defined CPUE as catch weight (kg) per unit vessel size (GT) per fishing day. Then we calculated the annual average CPUE for each fleet segment per FMA. To obtain the total annual catch for each fleet segment, we multiplied average CPUE with the estimated number of fishing days per year for that fleet segment and with the total gross tonnage of boats present in that fleet segment.

To calculate the total catch for each species per FMA, we multiplied CPUE estimates, the average fishing days in a year, the total gross tonnage in each fleet segment, and the species composition ratio (per fleet segment, per FMA). Total gross tonnage for each fleet segment was derived from frame survey results by summing vessel gross tonnage per fleet category per FMA (S3 Table).

### Life history parameters

Unreliable results from previous studies create a data gap for life history parameter values in Indonesia’s deep demersal fishery. Asymptotic length (L_inf_) is a key parameter and starting point in length-based assessments. In many recently published growth studies, L_inf_ for numerous species has been estimated by using age-length data to fit the von Bertalanffy growth equation (e.g., [19,20]). Estimation of the von Bertalanffy parameters, however, varies depending on the inputted age range [21]. Thus, many of these studies may be biased due to small sample sizes, samples from highly selective gear, aging error, or sourcing data from a single element of the fleet at a specific moment in time or from a specific location on the fishing grounds [22,23]. In fished populations, fast-growing young fish and slow-growing old fish are frequently over-represented in samples, leading to an underestimation of L_inf_ [23]. Additionally, in heavily fished ecosystems, researchers seldom have access to the rare surviving specimen at maximum length. These biases can cause underestimation of L_inf_, which poses a problem in the accuracy of stock assessments. An alternative approach to estimate life-history parameters is to estimate L_max_ as the largest specimen from a large sample of fish and use it to calculate other life-history parameter values based on known relationships between the parameters [24].

As a starting point for our length-based approach, we estimated the maximum attainable length (L_max_) for each species in the local population as the size of the largest recorded specimen in the catch (L_x-CODRS_). From L_max_, we calculated L_inf_, L_mat_, and L_opt_ (Table 1).

**Table 1:** Life history parameters used in this study for length-based stock assessments, including their definitions, calculation notes, and relevant reference.

Parameter	Definition	Calculation	Reference
L_max_	The maximum attainable length for each species in the local population	L_max_ = the size of the largest recorded specimen in the catch (L_x-CODRS_)	
L_inf_	The asymptotic length of the Von Bertalanffy growth equation	For all families, L_inf_ = 0.9 * L_max_	[25]
L_mat_	The length where 50% of the population gets mature for the first time	For Lutjanidae, L_mat_ = 0.59 * L_inf_	[26]
		For Epinephelidae, L_mat_ = 0.46 * L_inf_	[26]
		For all other families, we used L_mat_ = 0.5 * L_inf_	[27]
L_opt_	The length where an age group achieves maximum biomass in an unfished situation	For all families, L_opt_ = 1.33 * L_mat_	[28]

To further verify the updated life history parameters, we compared L_mat_ values from our calculation with maturity studies that determined the length at which 50% of the population matures (of the top 15 species in the catch). We chose L_mat_ estimates as a point of comparison because biological studies on maturation have been shown to be more robust than studies on L_inf_ [29]. We excluded studies that published values for length at first maturity. We compared L_mat_ values from areas with similar latitudes (15^o^ S– 15^o^ N); when not available, we included studies from other latitudes [30].

### Estimating SPR and defining additional length-based indicators

We defined spawning potential ratio (SPR) [31], as the current spawning stock biomass as a fraction of the spawning stock biomass in an unfished (pristine) situation [26]. Using length data to calculate SPR has been shown to be a viable approach for stock assessments [32,33]. We calculated SPR on a per-recruit basis from life-history parameters of total mortality (Z), fishing mortality (F), growth (K), and L_inf_. The instantaneous total mortality (Z) was estimated from catch length-frequency distribution with the equilibrium Beverton-Holt estimator, using Ehrhardt and Ault (1992) bias-correction, implemented through the function bheq2 of the *R Fishmethods* package [34,35].

As input for the procedure above, we used the length-frequency distribution of the total yearly catch of each species (all gears and all boat size categories combined). To obtain this length-frequency, we calculated an average length-frequency distribution per fleet segment, and we then combined length-frequency data with information on the fleet composition from the frame survey to estimate a length-frequency distribution of the total catch (all gears and boat size categories combined) over the most recent 365 days (March 23, 2019 –March 22, 2020).

The natural rate of mortality (M) was estimated using the Froese and Pauly (2000) empirical formula with L_inf_ as an estimated above, and ambient water temperature at fishing depth estimated at approximately 20 degrees Celsius [36]. With an asymptotic length for a snapper of about 80 cm, this resulted in an M of approximately 0.4, which aligned with other reported values from the literature [27]. The fishing mortality (F) was the difference between Z and M. We estimated the growth parameter (K) from L_opt_ and M and L_inf_, using the equation K = M*L_opt_/3*(L_inf_-L_opt_) [24].

### Length-based stock assessments

In data-poor fisheries, length-based assessment methods are a viable way to conduct stock assessments to determine fishery status and set management benchmarks [24,32,33,37,38]. Spawning potential ratio (SPR) as a reference point is not as data-demanding as other methodologies and can be useful to inform policy regarding the stock status. Decreasing SPR values indicates stock declines [39]. Froese et al. (2016) considered total population biomass (B) of 50% the pristine population biomass (B_o_) to be the lower limit reference point for stock size, minimizing the impact of fishing [40]. Using SPR and B/B_o_ estimates from our data set, the Froese et al. lower limit reference point correlates with an SPR of approximately 40%, not far from but slightly more conservative than the Wallace and Fletcher reference point [39,40]. Therefore, we chose an SPR of 40% as our reference point for "low risk" of stock collapse. After similar comparisons, we chose to consider SPR between 25% and 40% to represent a "medium risk" situation. We consider risk levels to be "high" at SPR values below 25%.

In addition to SPR, we used sizes of fish in the catch as an indicator of sustainability. An ideal target for catches is 0% immature fish [38], but a target of 10% or less is considered a reasonable indicator for sustainable harvesting of fish stocks [41,42]. Zhang et al. consider 20% immature fish in the catch as an indicator for a fishery at risk in their approach to an ecosystem-based fisheries assessment [43]. Results from a meta-analysis of many fisheries showed stock status over a range of stocks to fall below precautionary limits at 30% or more immature fish in the catch [42]. The fishery was considered at very great risk of collapse when more than 50% of the fish in the catch are immature and effort was high [40]. Thus, we considered risk levels to be "low" at levels of 10% or less immature fish in the catch, "medium" between 10% and 30%, and to be "high" at levels above 30% of immature fish in the catch.

We used the current exploitation level expressed as the percentage of fish in the catch below the optimum harvest size (L_opt_) as an indicator for fisheries status. This was the reciprocal value of the percentage of large mature fish above the optimum harvest size. We considered a proportion of 65% of the fish (i.e., the vast majority in numbers) in the catch below the optimum harvest size as an indicator for growth overfishing. We also considered a majority in the catch around or above the optimum harvest size as an indicator for minimizing the impact of fishing [40]. This indicator was achieved when less than 50% of the fish are below the optimum harvest size. We considered risk levels to be "low" at exploitation levels below 50%, "medium" between 50% and 65%, and "high" at levels of 65% or more.

We considered mega-spawners to be fish larger than 1.1 times the optimum harvest size (L_opt_) [38]. We considered a proportion of 30% or more "mega-spawners" in the catch to be a sign of a healthy population [38]. In contrast, lower proportions of mega-spawners led to concerns; proportions below 20% indicated a great risk to the fishery. Thus, we considered risk levels to be "low" at mega-spawner levels of 30% or more, "medium" between 20% and 30%, and "high" at levels below 20%.

We also used a static length indicator based on trade limit (minimum size accepted by fish traders for each species across all FMAs) and the size at maturity as an indicator of possible targeting of juveniles or for more sustainable targeting of mature fish that have spawned at least once. A trade limit that is well below the size at maturity indicates that the market generates demand for juvenile fish, exposing a larger proportion of the population to fishing pressure, and thereby making the population more vulnerable to over-fishing. We considered risk levels to be low when the trade limit > 1.1 × L_mat_; medium when the trade limit ≥ 0.9 × L_mat_; high when the trade limit < 0.9 × L_mat_.

## Results

### Indonesian deep demersal fishing fleet

Frame survey results showed a wide range of vessel sizes in the Indonesian deep demersal fisheries. Fishing boat sizes ranged from "nano" sized canoes of less than 1 GT, up to the larger vessels measuring close to 100 GT. The total deep demersal fishing fleet in Indonesia included an estimated 9,982 fishing boats (Table 2A), representing a total of more than 50,000 gross tons (GT) of combined vessel volume (Table 2B).

**Table 2:** (a) Summary of the deep demersal fishing fleet in Indonesia. (b) Combined total vessel volume (gross tonnage, GT) in the deep demersal fishing fleet in Indonesia. Data are by fleet segment (gear type, boat size, dedicated, seasonal) for all eleven Fishery Management Areas (FMAs) combined.

**Size Category**	**Fleet activity**	**(a) Number of Boats**					
		**Dropline**	**Longline**	**Gillnet**	**Trap**	**Mixgears**	**Total (GT)**
Nano	Dedicated	3085	533	0	63	327	4008
Nano	Seasonal	2169	316	0	20	678	3183
Small	Dedicated	500	104	16	353	294	1267
Small	Seasonal	402	31	2	0	5	440
Medium	Dedicated	165	259	18	262	102	806
Medium	Seasonal	92	8	6	0	0	106
Large	Dedicated	22	86	62	2	0	172
Large	Seasonal	0	0	0	0	0	0
	**Total**	**6435**	**1337**	**104**	**700**	**1406**	**9982**
**Size Category**	**Fleet Activity**	**(b) Total Gross Tonnage**					
		**Dropline**	**Longline**	**Gillnet**	**Trap**	**Mixgears**	**Total (GT)**
Nano	Dedicated	3653	804	0	203	947	5607
Nano	Seasonal	2959	356	0	27	915	4258
Small	Dedicated	3370	786	95	2195	1811	8257
Small	Seasonal	3340	220	14	0	32	3606
Medium	Dedicated	2846	5572	368	4988	1528	15302
Medium	Seasonal	1616	134	97	0	0	1847
Large	Dedicated	1325	5490	4685	65	0	11565
Large	Seasonal	0	0	0	0	0	0
	**Total**	**19110**	**13362**	**5259**	**7478**	**5233**	**50442**

We worked with 579 captains between October 2015 and March 2020 to implement the Crew Operated Data Recording System (CODRS) in Indonesia. These captains used drop lines (315 captains), bottom longlines (115 captains), mixgears (129 captains), traps (13 captains), and gillnet (7 captains). Through CODRS implementation, we obtained data from 8,914 fishing trips, which yielded 2,881,519 individual fish or 5,366.110 tons of catch. Vessels ranged from one to 115 GT in size. Recruitment of captains from the overall fleet into the CODRS program was not precisely proportional to the composition of the fleet in terms of vessel size, gear type, and the FMA where the boat typically operates. However, the fleet survey results allowed us to extrapolate catches from CODRS data to represent catches from the actual fleet.

### CODRS implementation and validation

Initial uptake to CODRS by fishers was met with hesitation and skepticism, both in being able to handle the additional workload and in ensuring data confidentiality. Establishing partnership with fish traders who provided the operational fishing cost helped leverage the CODRS by having the fish traders to be the ’champions’ of the system. Fish traders also helped us promote the system to the captains and crew. Subsequent uptake became easier as more and more captains signed up. Having an initial core group of willing captains paved the way to spread the system to other locations. We relied on captains or crew to share information with other prospective CODRS participants in the fishery. However, in one location, a fishing cooperative deliberately withheld information on other fishing cooperatives in the same fishery due to a long-standing rivalry. There were also technical challenges during the implementation of the system. For example, we needed to create smaller measuring boards to fit nano boats < 5 GT. We also added nets on the measuring board surface to prevent the slippage of fish specimens at sea. Some fishers were taking the batteries out of the GPS tracking units for their electronic appliances at home. In such situations, research technicians were critical in enforcing the rules of participation.

Captains and crews usually required one month of trial before they were familiar enough with the equipment and workflow before beginning data collection. During this period, research technicians were troubleshooting a variety of questions (e.g., issues with buttons on the camera) and gave examples on how to take proper photographs. Captains and crew were especially receptive to the GPS trackers as they believed it increased the safety of their vessel at sea. In fact, research technicians used the GPS tracker data twice during the study period to help locate vessels that had drifted at sea due to failed engines.

Anecdotal reports suggested that fishers highly benefited by the monetary compensation. Thus, having the contract contingent upon data quality helped research technicians to enforce the data collection best practices. Without the monetary compensation., given the workload, we suspect that compliance and data quality would be lower. Out of the 579 CODRS contracts, we terminated 60 due to poor data quality or by request from the captain.

**Fig 3:**
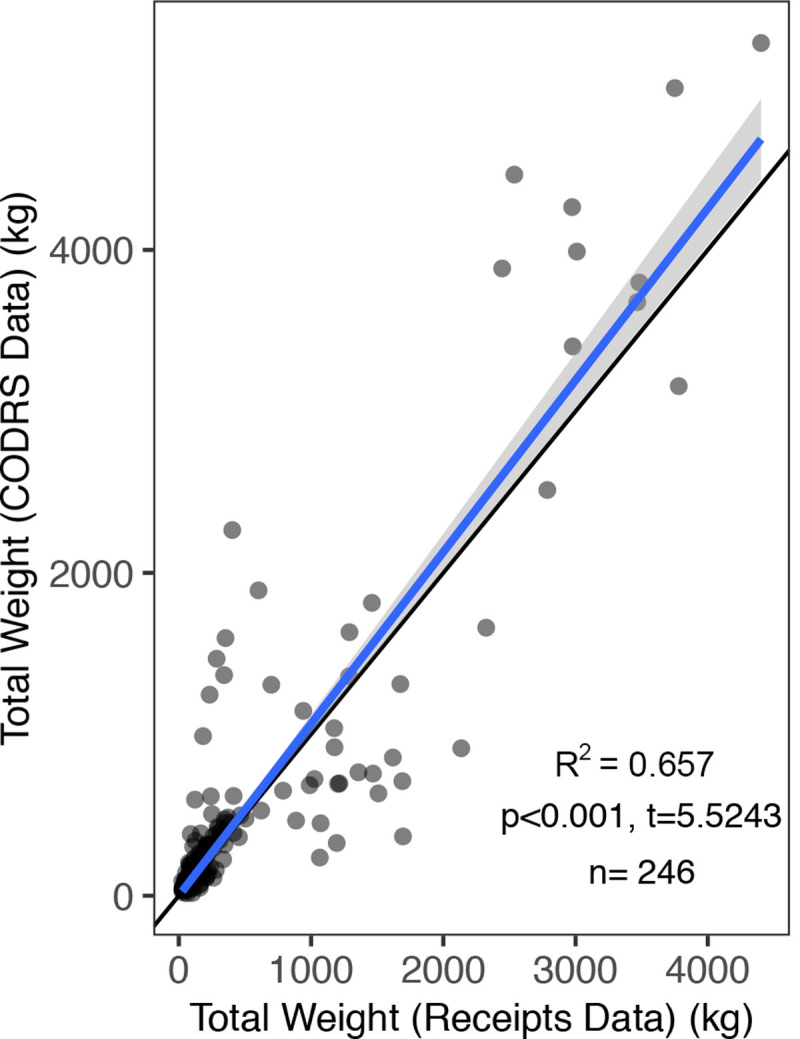
Total catch weight comparison between receipts and CODRS (Crew-Operated Data Recording System). The black line denotes 1:1 ratio between receipts and CODRS total weight; the blue line denotes fitted linear regression with 95% confidence interval in grey. R^2^ = 0.657; P-value and t value corresponds to the paired t-test results.

We used total weights from catch receipts (ledgers) as our control dataset to compare with CODRS results. In an experiment early on in this study, we obtained receipts from 41 captains with boats <30 GT, and from 3 captains with boats >30 GT. Because of the small sample size for large boats >30 GT, we did not use these data in our analysis. For boats <30 GT, we found significantly greater total catch weight per trip in CODRS than data collected from receipts (p < 0.001, t = 5.5243, R^2^ = 0.657). Our CODRS dataset also recorded more fish per catch than the receipts, and this became more pronounced as the catches got larger (Fig 3).

The annual cost to implement CODRS was approximately $3,600- $6,300 per vessel (depending on vessel size). The CODRS annual cost is substantially more expensive than that of logbooks ($42) but not observers ($2,700 per observer trip). CODRS is also a much less elaborate EM system than others, such as video camera monitoring ($11,200 to install per vessel and $4,481 annually per vessel) [15]. Cost of CODRS implementation covers hardware (camera, measuring board, training booklet, training posters, batteries, Spot Trace GPS tracker, memory card), payment to captains, Spot Trace GPS tracker annual subscription, and Network Area Storage (storage system for CODRS data).

### Catch characteristics

The deep demersal fisheries exploit more than 100 species of fish, but the top 50 species in the CODRS dataset together represent over 95% of all specimens recorded. Sample sizes ranging from 36,500 to 445,500 images were obtained for each of the top 15 species by weight, which together represented more than 75% of all recorded fish. The five most abundant species in CODRS samples represented well over 54% of all records. This group of five species included three snappers *(Lutjanus malabaricus*, *Pristipomoides multidens* and *Pristipomoides typus*), one small grouper (*Epinephelus areolatus*), and one croaker (*Atrobucca brevis*) (Table 3). One deep-water snapper species has not yet been scientifically described, so is noted here as ‘*Etelis* sp.’

**Table 3:** Top 15 species from CODRS (Crew Operated Data Recording System) ranked by weight (kg) and frequency from the deep demersal fisheries in Indonesia from across all fleet segments and FMAs between 2015 and 2020.

Species	Weight (kg)	% from total weight	n	% from n total
*Lutjanus malabaricus*	1,086,799	32.87	445,507	22.86
*Pristipomoides multidens*	628,551	19.01	336,004	17.24
*Atrobucca brevis*	228,964	6.92	301,169	15.46
*Pristipomoides typus*	218,707	6.61	174,598	8.96
*Aphareus rutilans*	177,964	5.38	64,105	3.29
*Etelis sp*.	159,074	4.81	42,779	2.20
*Epinephelus coioides*	135,627	4.10	31,201	1.60
*Lutjanus erythropterus*	127,950	3.87	100,635	5.16
*Lethrinus laticaudis*	104,999	3.18	59,888	3.07
*Lutjanus sebae*	94,438	2.86	48,374	2.48
*Pristipomoides filamentosus*	87,824	2.66	61,473	3.15
*Epinephelus areolatus*	77,913	2.36	192,951	9.90
*Paracaesio kusakarii*	65,677	1.99	37,296	1.91
*Etelis radiosus*	57,316	1.73	20,219	1.04
*Etelis coruscans*	54,935	1.66	32,416	1.66

To get a more thorough understanding of the catch characteristics of this fishery, we calculated the total catch by scaling up catches recorded by CODRS to represent the total fleet. The total catches showed unequal exploitation between gear types, FMAs, and vessel sizes. The total catch in 2019 was close to 90,000 tons, with approximately 47,000 tons caught by drop lines, more than 20,000 tons caught by bottom-set longlines, and 24,000 tons caught by a combination of gillnets, traps, and mixgears, respectively (S4 Table). The largest catches in 2019 (over 10,000 tons) were produced in FMAs 711 (12,458 tons), 712 (20,462 tons), 713 (10,274 tons), and 718 (12,692 tons). In other FMAs, catches ranged between 2,000 and 8,500 tons per year. After accounting for fleet composition, the top 20 species by volume represented close to 78% of the total catch, with more than 60% of the catch made up by eight species only, including seven large snappers and one large grouper (Table 4). Roughly two thirds of the total catch in 2019 was produced by vessels smaller than 10 GT.

**Table 4:** Total catch by volume (tons) of the top 20 species in the Indonesian deep demersal fisheries in 2019. Total catch was estimated based on CPUE, species distribution by fleet segment, average effort per fleet segment, and total vessel tonnage per FMA.

Species	Total Catch per FMA (tons)											Total catch (tons)
	571	572	573	711	712	713	714	715	716	717	718	
*Lutjanus malabaricus*	50	6	558	3552	7350	1092	75	161	33	59	4269	17205
*Pristipomoides multidens*	354	168	1589	2002	5525	957	271	495	76	852	886	13175
*Aphareus rutilans*	0	521	486	0	33	2026	313	3068	100	653	283	7483
*Etelis radiosus*	0	92	552	3	0	275	29	300	1657	722	13	3643
*Epinephelus coioides*	927	32	56	923	1095	134	83	12	76	56	219	3613
*Pristipomoides typus*	8	283	546	154	827	364	43	32	0	71	119	2447
*Etelis sp*.	0	257	74	0	0	548	271	673	180	305	99	2407
*Epinephelus areolatus*	88	69	77	993	725	234	24	31	8	52	55	2356
*Atrobucca brevis*	0	0	0	0	0	0	0	0	0	0	2091	2091
*Diagramma pictum*	1	11	48	1041	367	268	64	8	11	0	0	1819
*Etelis coruscans*	0	213	124	0	0	83	55	492	418	370	19	1774
*Caranx sexfasciatus*	71	147	49	37	122	561	242	185	167	105	62	1748
*Plectropomus maculatus*	0	2	0	1204	391	27	9	13	7	0	41	1694
*Aprion virescens*	0	663	20	47	142	104	151	168	122	12	110	1539
*Lutjanus erythropterus*	0	4	72	103	942	98	2	216	5	0	90	1532
*Pristipomoides filamentosus*	1	62	196	4	9	156	52	756	44	51	21	1352
*Lutjanus sebae*	1	0	72	326	296	221	115	15	4	0	276	1326
*Lethrinus olivaceus*	0	309	76	50	129	147	250	43	99	76	146	1325
*Caranx tille*	3	15	135	0	20	221	135	64	0	441	190	1224
*Caranx ignobilis*	10	391	26	0	51	130	187	26	309	8	81	1219

### Updating maximum length and other life-history parameters

**Fig 4:**
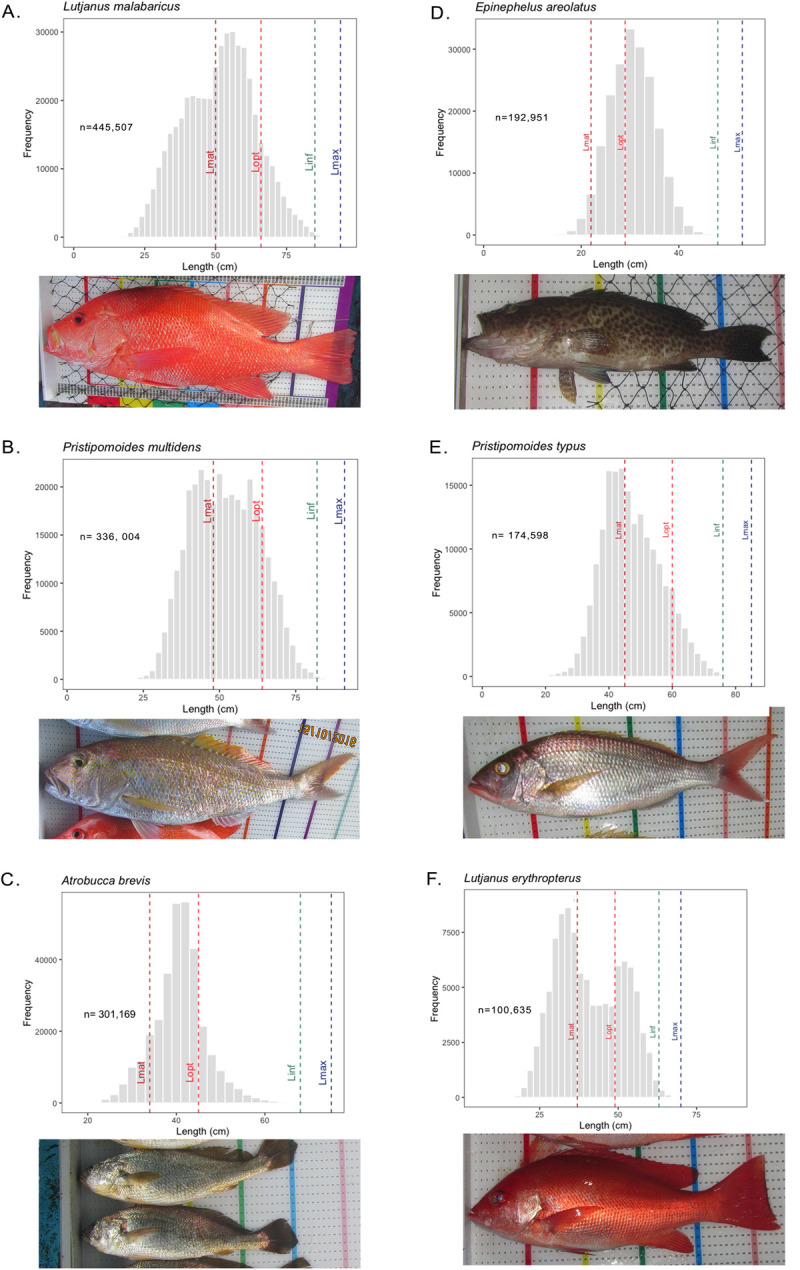
Length-frequency distributions of the six most frequently caught species in the Indonesian deep demersal fisheries (*Lutjanus malabaricus*, *Prisipomoides multidens*, *Pristipomoides typus*, *Epinephelus areolatus*, *Lutjanus erythropterus*, and *Atrobucca brevis*).

**Fig 5:**
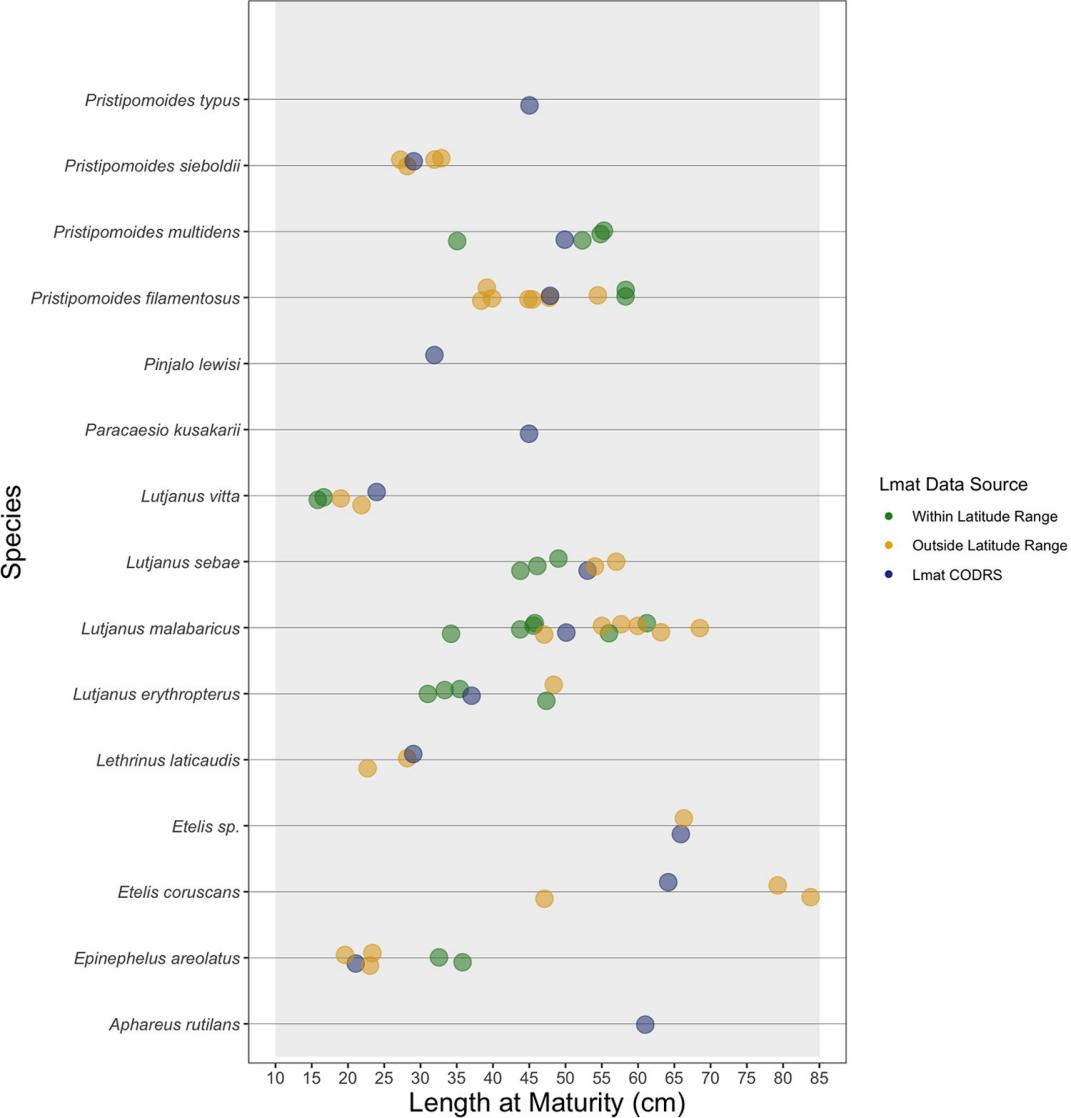
Length at maturity (L_mat_; cm) values for the top 15 species as well as *Etelis* sp.

Using CODRS images with sample sizes of at least 7,000 specimens per species, life-history parameters could be reliably updated for the top 50 species in CODRS dataset (Table 5), based on the maximum observed length in the catch (L_x-CODRS_). For several species, CODRS images provided values for maximum attainable lengths in Indonesian waters larger than previously reported. By treating L_max_ and L_inf_ as biological parameters instead of curve fitting parameters, we could estimate L_inf_ directly from L_max_ [28]. This method was supported by length-frequency distributions of each species, which demonstrated that specimens from L_x-CODRS_ used as L_max_ did not reflect anomalous fish (Fig 4). Photographs of specimen at L_x-CODRS_ form verifiable evidence of the lengths that these species can attain. Estimates of L_inf_ were then used to obtain estimates for L_mat_ and L_opt_ using life history invariants. In addition, our estimates for L_mat_ from life-history invariants result in values within the range of published values, with a few exceptions (Fig 5).

**Table 5:** Length-based life history parameters L_mat_, L_opt_, L_inf_, and L_max_ for the top 50 most abundant species in CODRS dataset from the deep demersal fisheries in Indonesia. L_max_ values were derived from L_x-CODRS_.

Fish Species	Lmat (cm)	Lopt (cm)	Linf (cm)	Lmax (cm)
*Lutjanus malabaricus*	50	66	85	94
*Pristipomoides multidens*	48	64	82	91
*Atrobucca brevis*	34	45	68	75
*Epinephelus areolatus*	22	29	48	53
*Pristipomoides typus*	45	60	76	85
*Lutjanus erythropterus*	37	49	63	70
*Lutjanus vitta*	23	31	39	43
*Aphareus rutilans*	64	85	108	120
*Pristipomoides filamentosus*	48	64	81	90
*Lethrinus laticaudis*	28	37	57	63
*Lutjanus sebae*	51	68	86	96
*Etelis sp*.	66	88	112	125
*Paracaesio kusakarii*	45	60	76	85
*Pristipomoides sieboldii*	30	40	51	57
*Diagramma pictum*	36	48	73	81
*Lutjanus timorensis*	34	45	58	65
*Etelis coruscans*	64	85	108	120
*Epinephelus coioides*	49	65	107	119
*Pinjalo lewisi*	31	41	52	58
*Gymnocranius grandoculis*	34	45	68	76
*Lethrinus lentjan*	25	33	50	55
*Carangoides chrysophrys*	36	48	72	80
*Etelis radiosus*	61	81	104	115
*Pinjalo pinjalo*	41	55	70	78
*Pomadasys kaakan*	29	39	58	64
*Lutjanus johnii*	48	64	81	90
*Caranx sexfasciatus*	40	53	81	90
*Aprion virescens*	57	76	96	107
*Plectropomus maculatus*	35	47	76	84
*Cephalopholis sonnerati*	25	33	54	60
*Paracaesio stonei*	37	49	63	70
*Caranx bucculentus*	34	45	68	75
*Wattsia mossambica*	27	36	54	60
*Epinephelus bleekeri*	33	44	71	79
*Lutjanus argentimaculatus*	51	68	86	95
*Lethrinus olivaceus*	44	59	87	97
*Lutjanus russelli*	28	37	48	53
*Lutjanus gibbus*	29	39	49	54
*Seriola rivoliana*	60	80	119	132
*Carangoides coeruleopinnatus*	31	41	62	69
*Lutjanus boutton*	18	24	30	33
*Plectropomus leopardus*	31	41	68	76
*Erythrocles schlegelii*	40	53	81	90
*Lutjanus bohar*	47	63	79	88
*Diagramma labiosum*	38	51	75	83
*Caranx ignobilis*	61	81	122	135
*Paracaesio gonzalesi*	29	39	49	54
*Caranx tille*	38	51	77	86
*Variola albimarginata*	20	27	44	49
*Elagatis bipinnulata*	49	65	98	109

### Length-based stock assessments

By utilizing SPR values and relative abundance by size-group (trade limit, immature, optimum length, mega-spawner), we characterized the risk levels of the 20 most abundant species in the fishery. Length-based stock assessments by FMA showed dangerously low SPR values and a high risk of overfishing in most FMAs for most target species in this fishery (S5 Table). This risk was pronounced for snapper species with large maximum size (Tables 6 and S6). There were differences in SPR values between FMA, although a high risk of overfishing was apparent for most major species in all FMAs. A yearly comparison of SPR indicated that FMA 573 (with major activity in the Timor Sea) showed signs of improvement, whereas FMA 712 (Java Sea) showed severe deterioration (Table 7). Such patterns of deterioration were evident in almost all FMAs in Indonesia (S7 Table).

**Table 6:** Risk levels of the 20 most abundant species in FMA 573 and FMA 712. Risk levels were determined using SPR values and relative abundance by size-group (trade limit, immature, optimum length, mega-spawner).

FMA	Species	Trade limit	%< Lmat	% < Lopt	% > Linf	% SPR
573	*Pristipomoides multidens*	high	high	high	high	high
573	*Pristipomoides typus*	high	high	high	high	high
573	*Lutjanus malabaricus*	high	high	high	high	high
573	*Epinephelus areolatus*	low	low	low	low	medium
573	*Pristipomoides sieboldii*	medium	low	high	high	high
573	*Pristipomoides filamentosus*	high	high	high	high	high
573	*Lutjanus erythropterus*	high	low	low	low	medium
573	*Paracaesio kusakarii*	high	medium	medium	medium	medium
573	*Lutjanus timorensis*	medium	medium	high	high	high
573	*Etelis coruscans*	high	high	high	high	high
573	*Pinjalo lewisi*	medium	medium	high	high	high
573	*Etelis sp*.	high	high	high	high	high
573	*Lutjanus sebae*	high	high	high	high	high
573	*Lutjanus vitta*	low	low	high	high	high
573	*Aphareus rutilans*	high	medium	medium	high	high
573	*Gymnocranius grandoculis*	high	low	low	low	low
573	*Etelis radiosus*	high	high	high	high	high
573	*Epinephelus morrhua*	high	low	high	high	high
573	*Parascolopsis eriomma*	low	low	low	low	low
573	*Glaucosoma buergeri*	medium	low	low	low	low
712	*Lutjanus malabaricus*	high	high	high	high	high
712	*Epinephelus areolatus*	low	low	low	low	medium
712	*Lutjanus erythropterus*	high	high	high	high	high
712	*Pristipomoides multidens*	high	high	high	high	high
712	*Lutjanus vitta*	low	high	high	high	high
712	*Diagramma pictum*	medium	high	high	high	medium
712	*Pinjalo pinjalo*	high	high	high	high	high
712	*Plectropomus maculatus*	medium	medium	low	low	low
712	*Epinephelus coioides*	medium	medium	high	medium	medium
712	*Carangoides chrysophrys*	low	high	high	high	high
712	*Lutjanus sebae*	high	high	high	high	high
712	*Lethrinus lentjan*	medium	low	low	low	medium
712	*Pristipomoides typus*	high	high	high	high	high
712	*Gymnocranius grandoculis*	high	high	high	high	high
712	*Lutjanus johnii*	high	high	high	high	high
712	*Epinephelus bleekeri*	high	low	low	low	low
712	*Carangoides coeruleopinnatus*	Unknown	Unknown	Unknown	Unknown	Unknown
712	*Carangoides gymnostethus*	medium	low	low	high	high
712	*Lutjanus russelli*	medium	low	medium	high	high
712	*Rachycentron canadum*	medium	medium	high	high	high

**Table 7:** Changes in SPR and in relative abundance by size group for the 20 most abundant species in 2019 from 2018 in the CODRS dataset in (a) FMA 573, (b) FMA 712. Unknown results indicated very low sample sizes.

FMA	Species	%< Lmat	% < Lopt	% > Linf	% SPR
573	*Pristipomoides multidens*	improving	improving	improving	improving
573	*Pristipomoides typus*	deteriorating	deteriorating	deteriorating	stable
573	*Lutjanus malabaricus*	deteriorating	improving	improving	improving
573	*Epinephelus areolatus*	deteriorating	deteriorating	deteriorating	improving
573	*Pristipomoides sieboldii*	deteriorating	deteriorating	deteriorating	improving
573	*Pristipomoides filamentosus*	deteriorating	improving	improving	improving
573	*Lutjanus erythropterus*	stable	deteriorating	improving	improving
573	*Paracaesio kusakarii*	improving	improving	improving	improving
573	*Lutjanus timorensis*	deteriorating	improving	deteriorating	improving
573	*Etelis coruscans*	improving	improving	improving	improving
573	*Pinjalo lewisi*	improving	improving	improving	improving
573	*Etelis sp*.	improving	improving	improving	improving
573	*Lutjanus sebae*	improving	improving	improving	improving
573	*Lutjanus vitta*	deteriorating	improving	improving	improving
573	*Aphareus rutilans*	deteriorating	improving	improving	improving
573	*Gymnocranius grandoculis*	improving	improving	improving	improving
573	*Etelis radiosus*	unknown	unknown	unknown	unknown
573	*Epinephelus morrhua*	improving	improving	improving	improving
573	*Parascolopsis eriomma*	stable	improving	improving	improving
573	*Glaucosoma buergeri*	improving	improving	improving	improving
712	*Lutjanus malabaricus*	deteriorating	deteriorating	deteriorating	deteriorating
712	*Epinephelus areolatus*	improving	improving	improving	improving
712	*Lutjanus erythropterus*	improving	improving	improving	improving
712	*Pristipomoides multidens*	deteriorating	deteriorating	deteriorating	deteriorating
712	*Lutjanus vitta*	deteriorating	deteriorating	deteriorating	deteriorating
712	*Diagramma pictum*	deteriorating	deteriorating	deteriorating	deteriorating
712	*Pinjalo pinjalo*	unknown	unknown	unknown	unknown
712	*Plectropomus maculatus*	unknown	unknown	unknown	unknown
712	*Epinephelus coioides*	unknown	unknown	unknown	unknown
712	*Carangoides chrysophrys*	deteriorating	deteriorating	deteriorating	deteriorating
712	*Lutjanus sebae*	deteriorating	stable	stable	stable
712	*Lethrinus lentjan*	deteriorating	improving	improving	deteriorating
712	*Pristipomoides typus*	deteriorating	deteriorating	deteriorating	deteriorating
712	*Gymnocranius grandoculis*	deteriorating	deteriorating	deteriorating	deteriorating
712	*Lutjanus johnii*	deteriorating	deteriorating	deteriorating	deteriorating
712	*Epinephelus bleekeri*	unknown	unknown	unknown	unknown
712	*Carangoides coeruleopinnatus*	improving	improving	stable	improving
712	*Carangoides gymnostethus*	unknown	unknown	unknown	unknown
712	*Lutjanus russelli*	unknown	unknown	unknown	unknown
712	*Rachycentron canadum*	unknown	unknown	unknown	unknown

## Discussion

### CODRS as a data recording and electronic monitoring system

CODRS data recorded more fish by weight than receipts; however, the variance around the 1:1 ratio was substantial. Receipts that indicated a total catch in the 10–500 kg range were associated with CODRS data indicating a catch of up to 1.5 metric tons. In the 500–2,500 kg per trip category, CODRS appeared to indicate a total catch that was around 50% lower than the figures indicated on the receipts. However, in the largest catches (> 2,500 kg) there was a high correlation between CODRS and the receipts.

Weight discrepancies could be explained by the generalization of length-weight relationships, potential bias in the photographs, fish being used as bait, eaten on-board, or sold directly to individual buyers (without any receipts) after being photographed and included in the CODRS data set. Also, there may have been some "cheating" by buyers, rigging weighing scales to record lower weights. Because of the prevalence of such activities, conducting the data collection onboard becomes crucial in getting the best catch estimates. Despite the uncertainty regarding the accuracy, CODRS is useful in the detailed effort data it records for each fishing trip. Using the CODRS dataset, researchers can match GPS coordinate dates from the tracking device to the date on catch photographs, verifying time, and general location of catches. These parameters help to standardize CPUE [44]. Researchers can also filter GPS coordinates to map specific fishing areas, determine the spatial distribution of fish species, analyze vessel dynamics, and determine management implications of different movement patterns [45–48].

The results showed that CODRS as an electronic monitoring (EM) system generated precise and detailed catch data for the Indonesian deep demersal fishery. CODRS is also a part of a global movement to transition from the conventional fishery-dependent data collection systems to more automated systems. However, even though the data recording process in CODRS is semi-automated with cameras, the implementation of the system still relies heavily on human resources for intensive data analysis, training, and monitoring of captains and crews. For example, after research technicians received data from fishers, the technicians always needed to provide feedback on the data quality. Constant monitoring as a form of feedback was necessary to ensure compliance with the monitoring protocol [49]. Thus, pre- and post-data collection efforts remain high and unavoidable, given the multi-species and disperse nature of the fisheries.

In the context of the CODRS program, the most critical issues that we had to address were: (i) captains needed to take photographs of their entire catch and not just a portion (i.e., by excluding sharks and other bycatch) or their perception of the targeted catch; (ii) captains or their designated crew needed to take photographs of sufficient quality (e.g., pictures were sometimes blurry, or the camera was not appropriately angled); and (iii) captains needed to position fish on the measuring board properly. If these problems were not identified by the trained technicians, it would have led to poor data quality and misrepresentation of the catch.

### The future of CODRS

To be a useful monitoring tool, scaling up the CODRS and adopting it as a long-term system is crucial. Manual and automatic species identification have been tested in several multi-species fisheries [14,16,50]. In manual species identification, reviewers watch video footage of the catch and identify the species. Both CODRS and other similar systems face the same problem of blurry imagery and human error in species identification. Automatic identification, on the other hand, has showed promising results in some fisheries. One example is the Catch Meter, a computer vision machine tested on Norwegian fishing vessels, which identified seven flatfish species with 99.8% accuracy. However, the device (a camera stationed on top of a conveyor belt carrying fish) is 3.5 meters long and not feasible to implement in the Indonesian deep demersal fishing vessels. We expect that image analysis automation through artificial intelligence will expedite the species identification process and remove many of the technical barriers to data analysis [50]. Although still in development, these technologies should soon be available, and CODRS would be improved significantly, both in accuracy and cost. In addition, improvements in photo or video species recognition devices can promote and engage participatory data collection as the automation will significantly reduce the workload of fishers and captains.

The cost of deployment and implementation may be a hurdle to upscaling CODRS or maintaining the system for long periods of time. Even though the initial price to implement the system could be offset by the amount of data obtained and subsequent management implications, without proper funding strategies, government agencies might not choose to adopt it. Especially since compliance may be tied to the monetary compensation, even with automated species recognition, CODRS will be more expensive than logbooks. However, given the amount of data obtained from CODRS and its accuracy, the value of this method far exceeds that of other methods. Logbooks, observers, and CODRS all require fishers or observers to provide unbiased and accurate data voluntarily, so this caveat is not exclusive to one method over another.

In addition to providing catch and effort data, CODRS as a collaborative system could act as a precursor to co-management of a fishery [51,52]. Collaborative approaches to fisheries management have gained traction in recent years as a potential solution to data-poor and open-access tropical fisheries such as those found across Indonesia [53]. This approach relies on the sharing of power and knowledge between policy-makers, researchers, and resource-users [54]. A potential next step would be to expand the CODRS network as a data-sharing platform to initiate discussion on fishery trends and challenges among fishers and fish traders. Success has been shown in similar fisheries to this one, which fostered collaboration and data collection for stock assessments [51,55].

### Species composition and trade significance

CODRS data allowed researchers to discern catch composition at the species level. The most important species in the catch by volume in 2019 was the Malabar Snapper (*Lutjanus malabaricus*), yielding close to 17,000 tons or 19% of the total catch in the fishery. Malabar snapper is sometimes mixed in the trade (especially in trade of fillets) with other species such as the Timor Snapper (*Lutjanus timorensis*) and the Mangrove Snapper (*Lutjanus argentimaculatus*). Production of Timor Snapper and Mangrove Snapper, however, is not very high in Indonesia. Two more snapper species of the genus Lutjanus, the Crimson Snapper (*Lutjanus erythropterus*) and the Red Emperor (*Lutjanus sebae*), are usually traded separately and both make the list of top 20 species with more than 1,500 tons of Crimson Snapper and close to 1,300 tons of Red Emperor. The above five species of Lutjanids, which are all red in color and are therefore sometimes traded as "Red Snapper", together accounted for close to 22,000 tons and close to 25% of the total deep demersal fisheries catch in 2019.

The second most important species in the catch was the Goldband Snapper (*Pristipomoides multidens*), which yielded over 13,000 tons in 2019. This species is commonly mixed in the trade with the Sharptooth Jobfish (*Pristipomoides typus*) of which close to 2,500 tons was landed in 2019. One more look-alike species, the Opakapaka (*Pristipomoides filamentosus*), is usually traded separately and was also in the list of top 20 species with more than 1,350 tons landed in 2019. These three closely resembling species of the genus *Pristipomoides*, all reddish in color including one with gold colored bands, totalled around 17,000 tons or close to 20% of the deep demersal fisheries catch in 2019.

A third important group of red colored snappers (Lutjanidae) includes the Rusty Jobfish or Lehi (*Aphareus rutilans*), the Ruby Snappers or Ehu (*Etelis carbunculus* and *Etelis* sp.), the Pale Snapper (*Etelis radiosus*) and the Flame Snapper or Onaga (*Etelis coruscans*). Together these large red colored snappers accounted for another 15,000 tons or 17% of the catch in 2019. *Etelis carbunculus* is a rare (and smaller) species in Indonesia, while its larger cousin has not yet been scientifically described. The Pale Snapper, *Etelis radiosus*, is often combined in the trade with *Etelis* sp. under "Ruby Snapper" or "Ehu", which is often incorrectly labelled as *Etelis carbunculus*.

There are several more red or reddish colored snappers in the deep demersal fisheries catch, such as *L*. *bitaeniatus*, *L*. *bohar*, *L*. *gibbus*, *L*. *johnii*, *L*. *russeli*, and *L*. *lemniscatus*. The trade name "Red Snapper" is clearly not useful in identifying the species or even the genus of the fish. Outside the genus of the Lutjanids, red colored snappers with common name Slender Pinjalo (*Pinjalo lewisi*) often get mixed in the trade with the above-mentioned Crimson Snapper (*L*. *erythropterus*), while Chinaman Snapper (*Symphorus nematophorus*) is usually filleted and cut into "portions" and sometimes sold as Malabar Snapper. More species from other genera are mixed in the snapper trade, especially in "skin off" fillets and "portions", where skin color is of no consequence. This includes one more snapper species from the list of top 20 species, the Green Jobfish (*Aprion virescens*), contributing well over 1,500 tons to the total catch in 2019, as well as other poorly known species such as the Saddle-back Snapper *Paracaesio kusakarii* and other *Paracaesio* spp. Altogether, the above mentioned snapper species, many but not all of them red in color, contributed more than 60,000 tons to the total deep demersal fisheries catch in 2019.

Non-snapper species in the top 20 of deep demersal catches include two species of groupers, the large growing Orange Spotted Grouper or Estuary Cod (*Epinephelus coioides*) and the smaller Areolate Grouper or Square Tail Rock Cod (*Epinephelus areolatus*). Both species contributed around 7,700 tons to the deep demersal catch. Two major species of emperors, the Long Nose Emperor (*Lethrinus olivaceus*) and the Blue-lined Emperor (*Gymnocranius grandoculis*) jointly contributed close to 2,500 tons to the catch. A third species of emperor, the Grass Emperor (*Lethrinus laticaudis*) was important locally in the Arafura Sea fisheries, where the Orange Croaker (*Attrobuca brevis*) and Black Jewfish (*Protonibea diacanthus*) were also abundant in local catches. Jacks, trevallies and grunts added almost 6,000 tons of mostly lower value species. The top 20 species in the catch in terms of volume together accounted for almost 71,000 tons or close to 80% of the entire catch of our 100 target species.

### The validity of the updated life history parameters

We noted a lack of consistency in L_mat_ values across studies over the range of our target species. For example, L_mat_ studies of *P*. *filamentosus* from latitudes near the equator tended to estimate larger values than values published in studies conducted at higher latitudes [56–58]. However, the opposite trend occurred in L_mat_ values for *L*. *sebae*, *L*. *malabaricus*, and *L*. *erythropterus* [19,59,60]. L_mat_ estimates from our methodology for *P*. *sieboldii*, *P*. *filamentosus*, *L*. *sebae*, *L*. *malabaricus*, *L*. *erythropterus*, and *Epinephelus areolatus* were somewhere in the middle of previously published ranges. Our L_mat_ estimates of *P*.*multidens* and *Etelis* sp. were lower than previous estimates in similar latitudes [5,59,60]. Finally, our L_mat_ estimates of *Lutjanus vitta* and *Lethrinus laticaudis* were larger than previous estimates. Values calculated from CODRS compared to those from the literature that were either inside or outside the latitude range of where they were caught in this study.

The broad range in published values for L_mat_ within species highlights the need for caution before referring to any particular value or study as well as a need for establishing local estimates, because changes in estimates for L_mat_ will directly affect conclusions from stock assessments. For example, L_mat_ for *P*. *multidens* had the largest range of values from the literature, with 35 cm being the lowest [61] and 61 cm the highest [59]; ours was 50 cm. Mees [56] estimated L_mat_ for *P*. *filamentosus* in the Seychelles (58 cm) with samples encompassing a wide size range and large sample size. But then Ralston and Miyamoto [57] estimated L_mat_ at 44 cm from a very limited sample size. None of the previous research can represent the L_mat_ of *P*. *filamentosus* in Indonesian waters, however, as our estimate is between the values proposed by the two studies. L_mat_ values for *L*. *laticaudis* were 22 cm (female) and 18 cm (male) [62]. These values were lower than our L_mat_ estimate; however, they originated from Shark Bay, Western Australia, which is outside the latitudinal range of our catches. The lack of previous maturity research on these species leads to high uncertainties in estimating plausible ranges for L_mat_.

While reviewing literature, statistics, and trading reports, determining the validity of published data remained a challenge due to potential species misidentification. *Aphareus rutilans*, for example, has sometimes been traded as *Aphareus furca*, which has a much smaller L_max_ than *A*. *rutilans*, and predominantly lives in shallower habitat. Only after better understanding the fisheries (fishing area, depth, gear type, and species distribution) could we infer that what has been recorded as *A*. *furca* prior to the present study was actually *A*. *rutilans*. In another example, differences between *Etelis carbunculus* and *Etelis* sp. have only recently been reported. The latter species grows more than twice as long as the former, is an important species in the deep demersal fisheries, but has yet to be scientifically described. Literature from before 2015 refers only to *E*. *carbunculus* with life-history parameter values reported that could only have come from *Etelis* sp. Numerous publications from before 2015 also misidentified the most common snapper in the deep demersal fisheries, *Lutjanus malabaricus* as *Lutjanus sanguineus*, a species that does not even occur in Indonesia. Such misidentifications of species have in the past resulted in many misunderstandings related to the Indonesian deep demersal fisheries, but with the image-based CODRS approach, our data can always be verified by returning to the photographs.

We also found a disparity between available information in the literature and abundance of the species in the catch. Hardly any studies were available for *Pristipomoides typus*, the third most abundant snapper species in CODRS dataset. *P*. *typus* is often mixed by traders with *P*. *multidens* due to their morphological similarities. However, *P*. *multidens* grows to a larger maximum size than *P*. *typus* and thus has different life-history parameters. Very few studies were available on life-history parameters or other biological characteristics of the most abundant grouper in these fisheries, *Epinephelus areolatus*. Maturity studies were lacking for *Aphareus rutilans*, *Pinjalo lewisi*, and *Paracaesio kusakarii*, despite their prevalence in the catches. These disparities highlighted a data gap in the literature that would have hampered our understanding of these important deep demersal fisheries without the new information obtained from the CODRS approach.

### Management implications

Length-based stock assessments showed high risks of overfishing for most target species in the deep demersal fisheries in Indonesia, especially for snapper species with large L_max_. All major target species of snapper showed a steep decline in catch volume and size where they are most vulnerable to fishing. This steep decline indicates high fishing mortality for the vulnerable size classes. For several snapper species, fishers consistently targeted and landed catches well below L_mat._ Almost all the larger species were harvested well below L_opt_. Most grouper species, on the other hand, had already reached or passed L_opt_ when they were caught by the deep demersal fisheries in Indonesia.

The increased granularity in CODRS data allows these findings to be directly applicable to managers in each FMA to determine each species’ importance to the fishing industry and risk of overfishing. We found common or trade names that were previously utilized were not adequate to be a basis for management strategies. For example, we noted differences in over-exploitation risk for species marketed under the same trade name (e.g., *L*. *malabaricus* and *L*. *timorensis* as ’red snapper’). Because of the differences in L_mat_, the same plate-sized trade limit would have a higher impact on *L*.*malabaricus* than on *L*. *timorensis*. We also noted differences in over-exploitation risks and top species among FMAs, making homogeneous species-based management not possible. Our results allow managers in each FMA to focus on strategies for the dominant and most vulnerable species in the area. For example, *L*. *erythropterus* has a high risk of over-exploitation based on all metrics and is a dominant catch species in FMA 712. However, *L*. *erytropterus* has a low and medium risk of over-exploitation based on the length-based history parameters and SPR, respectively, and is a less frequently caught species in FMA 573.

Based on the vessel and catch characteristics, we identified two management recommendations that can improve sustainability of the fisheries. Our first recommendation is to reduce market demand for juvenile fish. Traders should adjust their purchase orders, which usually specify quantities of fish by size bracket, to avoid sourcing juvenile fish. Some experimental evidence exists that, at least for some species, reduced demand for juveniles will result in a reduction of fishing mortality [63]. In consideration of this recommendation, one of Indonesia’s Fishery Improvement Projects (FIP) that is moving towards certification to Marine Stewardship Council standards required its participants to avoid sourcing of juvenile snappers. Furthermore, market preference for small size classes (i.e., plate-size fillets) could be reduced through awareness campaigns that clarify to the public that this preference impairs fisheries sustainability. In addition, the government could consider imposing a legal minimum size corresponding to the size at first maturity. Since it will be difficult to enforce this measure on remote landing sites, the government may consider to apply this measure to processing plants and cold storage facilities only. This measure will offer partial protection to juvenile fish and allow a larger proportion of recruits to contribute to the spawning stock.

Our second recommendation is to cap fishing effort at the current level and explore incentives for effort reductions, either by gradually reducing the number of active licenses or shortening the fishing season. These measures can be implemented through Indonesia’s licensing system, and for larger boats (> 30 GT), Indonesia’s Vessel Monitoring System allows for efficient enforcement of this measure. For fishing vessels that are smaller than 10 GT, which are not subject to the licensing system, an area-based approach (Marine Protected Areas) and/or co-management schemes may be effective as a means to control fishing effort [64,65]. Continuous monitoring of trends in the various size-based indicators will show the direction these fisheries are heading and the effects of any fisheries management measures in future years.

## Conclusions

A multi-species data collection program of the scale of this study has never been documented before in tropical deep demersal fisheries. Our Crew Operated Data Recording System (CODRS) proved to be an efficient and effective system to collect high-resolution catch and effort data, including species and size distribution of catches, fishing locations, and detailed information on fleet size, gear types, and fleet dynamics. Data issues caused by offloading at sea, reporting of "commercial" catch only instead of the total catch, catch consumed by the crew or used as bait, did not affect CODRS data, whereas these would have had led to data bias in port sampling programs.

The vast quantity and quality of verifiable image-based length measurements by species in the catch enabled us to update important life-history parameters, perform length-based stock assessments, and ultimately generate actionable management advice. Catch characteristics and stock assessment results using CODRS data showed differences in the sustainability of the species across the FMAs. The fishing industry can influence catch sizes by establishing larger trade sizes for snapper species. This initiative may be complemented by government regulations on minimum allowable sizes for all species. The government must also take action to cap or reduce fishing effort. Especially for unlicensed smaller-scale fishers, which contributes to most of the catches in the fishery, co-management schemes and/or no-take closures may be essential tools in steering the fisheries to a healthy status.

## Supporting information

S1 Table The length-weight relationship (a and b values) and conversion factors from fork length (FL) or standard length (SL) to total length (TL) for the top species in the deep demersal fishery. (DOCX)

S2 Table Average number of active fishing days per year for different gear types and different boat size categories (nano, small, medium, and large) in the deep demersal fishing fleet in Indonesia in 2019 across all FMAs. (CSV)

S3 Table Number of vessels and total vessel gross tonnage (GT) per fleet segment and FMA based on the frame survey. (XLS)

S4 Table Total catch by fleet segment (in metric tons) of the combined 100 target species in the Indonesian deep demersal fisheries, for all FMAs combined with “mixed gear” representing operations combining any of the other 4 gear types. (XLSX)

S5 Table Spawning potential ratio (SPR) values for the 20 most-abundant species in the Indonesian deep demersal fisheries catch in 2019. “NA” indicates very low sample sizes.
(XLSX)

S6 Table Risk levels of the 20 most-abundant species (ranked by abundance) in FMAs 571, 572, 711, 713, 714, 715, 716, 717, and 718. Unknown results indicated very low sample sizes.
(XLSX)

S7 Table Changes in spawning potential ratio (SPR) and relative abundance by size group for the 20 most-abundant species in FMAs 571, 572, 711, 713, 714, 715, 716, 717, and 718 between the 2019 and 2018 CODRS dataset. Unknown results indicated very low sample sizes.
(XLSX)
