# Supplementary material for: A crew-operated data recording system for length-based stock assessment of Indonesia’s deep demersal fisheries
Source: PLoS One. 2022 Feb 25;17(2):e0263646. doi: 10.1371/journal.pone.0263646 (PMC8880757; doi:10.1371/journal.pone.0263646)
Supplement: S1 Table — (DOCX) [file pone.0263646.s001.docx]

| **Species** | **W = a * L^b** | | **Sample a and b information** | | **Sample L Range** | | **Sample L Type** | **Conversion Factor** | **Reference** |
| --- | --- | --- | --- | --- | --- | --- | --- | --- | --- |
|  | **a** | **b** | **Sample n** | **Sample Area** | **Lmin** | **Lmax** | **TL/FL/SL** | **L to TL** |  |
| *Aphareus rutilans* | 0.015 | 2.961 | 73 | North Marianas | 42 | 98 | FL | 0.851 | [1] |
| *Aprion virescens* | 0.023 | 2.886 | 121 | New Caledonia | 22 | 88 | FL | 0.882 | [2] |
| *Etelis carbunculus* | 0.017 | 3.010 | 1221 | Hawaii | 19 | 64 | FL | 0.918 | [3] |
| *Etelis sp.* | 0.022 | 2.950 | 1814 | Vanuatu | 20 | 100 | FL | 0.918 | [4] |
| *Etelis radiosus* | 0.056 | 2.689 | ?? | Vanuatu | 40 | 89 | FL | 0.882 | [5] |
| *Etelis coruscans* | 0.041 | 2.758 | 1283 | Vanuatu | 20 | 90 | FL | 0.800 | [5] |
| *Pristipomoides multidens* | 0.020 | 2.944 | 2941 | Vanuatu | 26 | 70 | FL | 0.893 | [5] |
| *Pristipomoides typus* | 0.014 | 2.916 | ?? | Indonesia | 9 | 67 | TL | 1.000 | [6] |
| *Pristipomoides filamentosus* | 0.038 | 2.796 | 1531 | Hawaii | 16 | 80 | FL | 0.893 | [3] |
| *Pristipomoides sieboldii* | 0.022 | 2.942 | 329 | Hawaii | 23 | 44 | FL | 0.874 | [3] |
| *Pristipomoides argyrogrammicus* | 0.013 | 3.140 | 23 | North Marianas | 23 | 33 | FL | 0.868 | [7] |
| *Pristipomoides zonatus* | 0.041 | 2.833 | 189 | Hawaii | 31 | 50 | FL | 0.868 | [3] |
| *Pristipomoides flavipinnis* | 0.030 | 2.825 | 1660 | Vanuatu | 20 | 61 | FL | 0.872 | [5] |
| *Lutjanus bitaeniatus* | 0.014 | 2.980 | Based on body shape |  | N/A | N/A | FL | 0.983 | [8] |
| *Lutjanus argentimaculatus* | 0.034 | 2.792 | 365 | New Caledonia | 6 | 68 | FL | 0.983 | [9] |
| *Lutjanus bohar* | 0.016 | 3.059 | 510 | New Caledonia | 4 | 75 | FL | 0.948 | [2] |
| *Lutjanus malabaricus* | 0.009 | 3.137 | 965 | Vanuatu | 27 | 62 | FL | 1.000 | [5] |
| *Lutjanus sebae* | 0.009 | 3.208 | 1116 | Australia | 20 | 70 | FL | 0.959 | [10] |
| *Lutjanus timorensis* | 0.009 | 3.137 | Copied from L, malabaricus, same body shape. |  | N/A |  | FL | 0.993 |  |
| *Lutjanus gibbus* | 0.015 | 3.091 | 501 | New Caledonia | 16 | 41 | FL | 0.929 | [9] |
| *Lutjanus erythropterus* | 0.024 | 2.870 | 255 | Australia | ?? | ?? | FL | 1.000 | [11] |
| *Pinjalo lewisi* | 0.014 | 2.970 | Based on body shape. |  | N/A |  | FL | 0.959 | [8] |
| *Pinjalo pinjalo* | 0.014 | 2.970 | Based on body shape. |  | N/A |  | FL | 0.912 | [8] |
| *Lutjanus johnii* | 0.020 | 2.907 | Copied from L. russelli, same body shape. |  | N/A |  | FL | 0.957 |  |
| *Lutjanus russelli* | 0.020 | 2.907 | 200 | New Caledonia | 10 | 37 | FL | 0.957 | [9] |
| *Lutjanus lemniscatus* | 0.020 | 2.907 | Copied from L. russelli, same body shape. |  | N/A |  | FL | 0.957 |  |
| *Lutjanus vitta* | 0.017 | 2.978 | 1013 | New Caledonia | 6 | 39 | FL | 0.967 | [9] |
| *Lutjanus boutton* | 0.034 | 3.000 | ?? | Papua New Guinea | ?? | ?? | FL | 0.962 | [12] |
| *Lutjanus rivulatus* | 0.008 | 3.260 | 12 | New Caledonia | 16 | 76 | FL | 0.972 | [2] |
| *Lipocheilus carnolabrum* | 0.149 | 2.488 | 88 | Vanuatu | 30 | 68 | FL | 0.923 | [5] |
| *Symphorus nematophorus* | 0.015 | 3.046 | 41 | New Caledonia | 45 | 92 | FL | 0.962 | [2] |
| *Paracaesio gonzalesi* | 0.020 | 3.050 | Based on body shape. |  | N/A |  | FL | 0.931 | [8] |
| *Paracaesio xanthura* | 0.023 | 3.000 | 2 | Papua New Guinea | 19 | 21 | SL | 0.863 | [12] |
| *Paracaesio kusakarii* | 0.011 | 3.135 | 29 | Vanuatu | 20 | 62 | FL | 0.890 | [5] |
| *Paracaesio stonei* | 0.024 | 2.960 | Based on body shape. |  | N/A |  | FL | 0.890 | [8] |
| *Saloptia powelli* | 0.008 | 3.175 | 47 | North Marianas | 33 | 47 | FL | 1.000 | [5] |
| *Cephalopholis miniata* | 0.026 | 2.864 | 77 | New Caledonia | 23 | 45 | TL | 1.000 | [9] |
| *Cephalopholis sexmaculata* | 0.027 | 3.000 | ?? | Papua New Guinea | ?? | ?? | SL | 0.792 | [12] |
| *Cephalopholis sonnerati* | 0.015 | 3.058 | 67 | New Caledonia | 24 | 50 | TL | 1.000 | [9] |
| *Cephalopholis igarashiensis* | 0.049 | 2.748 | 10 | North Marianas | 23 | 41 | FL | 1.000 | [7] |
| *Epinephelus latifasciatus* | 0.010 | 3.088 | 179 | Kuwait | ?? | ?? | TL | 1.000 | [13] |
| *Epinephelus radiatus* | 0.061 | 2.624 | Copied from E. morrhua, same body shape. |  | N/A |  | FL | 1.000 |  |
| *Epinephelus morrhua* | 0.061 | 2.624 | 251 | Vanuatu | 26 | 80 | FL | 1.000 | [4] |
| *Epinephelus poecilonotus* | 0.061 | 2.624 | Copied from E. morrhua, same body shape. |  | N/A |  | FL | 1.000 |  |
| *Epinephelus areolatus* | 0.011 | 3.048 | 268 | New Caledonia | 6 | 43 | FL | 0.979 | [2] |
| *Epinephelus bleekeri* | 0.009 | 3.126 | 11 | Thailand | 14 | 27 | TL | 1.000 | [14] |
| *Epinephelus miliaris* | 0.026 | 3.000 | ?? | Papua New Guinea | ?? | ?? | SL | 0.778 | [12] |
| *Epinephelus bilobatus* | 0.014 | 2.990 | Copied from E. maculatus, same body shape. |  | N/A |  | TL | 1.000 |  |
| *Epinephelus malabaricus* | 0.013 | 3.034 | 198 | New Caledonia | 9 | 128 | TL | 1.000 | [9] |
| *Epinephelus coioides* | 0.011 | 3.084 | 41 | New Caledonia | 7 | 111 | TL | 1.000 | [9] |
| *Epinephelus chlorostigma* | 0.015 | 2.940 | ?? | Seychelles | ?? | ?? | FL | 1.000 | [15] |
| *Epinephelus retouti* | 0.027 | 3.000 | Copied from C. sexmaculata, same body shape. |  | N/A |  | SL | 0.792 |  |
| *Epinephelus heniochus* | 0.061 | 2.624 | Copied from E. morrhua, same body shape. |  | N/A |  | FL | 1.000 |  |
| *Epinephelus stictus* | 0.027 | 3.000 | Copied from C. sexmaculata, same body shape. |  | N/A |  | SL | 0.792 |  |
| *Epinephelus epistictus* | 0.009 | 3.126 | Copied from E. bleekeri, same body shape. |  | N/A |  | TL | 1.000 |  |
| *Epinephelus multinotatus* | 0.017 | 2.964 | 54 | Kuwait | ?? | ?? | TL | 1.000 | [13] |
| *Epinephelus undulosus* | 0.015 | 2.940 | Copied from E. chlorostigma, same body shape. |  | N/A |  | FL | 1.000 |  |
| *Epinephelus amblycephalus* | 0.012 | 3.057 | Copied from E. polyphekadion, same body shape. |  | N/A |  | TL | 1.000 |  |
| *Hyporthodus octofasciatus* | 0.106 | 2.560 | Copied from E. tukula, same body shape. |  | N/A |  | TL | 1.000 |  |
| *Plectropomus maculatus* | 0.016 | 3.000 | 2 | Australia | ?? | ?? | FL | 1.000 | [16] |
| *Plectropomus leopardus* | 0.012 | 3.060 | 191 | New Caledonia | 11 | 91 | FL | 0.976 | [2] |
| *Variola albimarginata* | 0.012 | 3.079 | Copied from V. louti, same body shape. |  | N/A |  | FL | 0.876 |  |
| *Lethrinus atkinsoni* | 0.018 | 3.057 | 2038 | New Caledonia | 4 | 46 | FL | 0.944 | [2] |
| *Lethrinus lentjan* | 0.020 | 2.986 | 380 | New Caledonia | 7 | 45 | FL | 0.955 | [2] |
| *Lethrinus laticaudis* | 0.020 | 2.986 | Copied from L. lentjan, same body shape. |  | N/A |  | FL | 0.955 |  |
| *Lethrinus nebulosus* | 0.019 | 2.996 | 2980 | New Caledonia | 4 | 70 | FL | 0.935 | [2] |
| *Lethrinus olivaceus* | 0.029 | 2.851 | 135 | New Caledonia | 23 | 73 | FL | 0.927 | [2] |
| *Lethrinus amboinensis* | 0.029 | 2.851 | Copied from L. olivaceus, same body shape. |  | N/A |  | FL | 0.908 | [2] |
| *Lethrinus rubrioperculatus* | 0.013 | 3.108 | 661 | New Caledonia | 17 | 40 | FL | 0.908 | [5] |
| *Wattsia mossambica* | 0.040 | 2.824 | 39 | Vanuatu | 24 | 48 | FL | 0.962 | [5] |
| *Gymnocranius grandoculis* | 0.032 | 2.885 | 243 | New Caledonia | 16 | 69 | FL | 0.931 | [2] |
| *Gymnocranius griseus* | 0.032 | 2.885 | Copied from G. grandoculus, same body shape. |  | N/A |  | FL | 0.930 |  |
| *Carangoides coeruleopinnatus* | 0.032 | 2.902 | 19 | New Caledonia | 12 | 31 | FL | 0.881 | [2] |
| *Carangoides fulvoguttatus* | 0.033 | 2.808 | 54 | New Caledonia | 16 | 81 | FL | 0.906 | [2] |
| *Carangoides malabaricus* | 0.023 | 3.020 | 39 | China | 15 | 44 | FL | 0.861 | [17] |
| *Carangoides chrysophrys* | 0.027 | 2.902 | 130 | New Caledonia | 13 | 60 | FL | 0.894 | [2] |
| *Carangoides gymnostethus* | 0.046 | 2.746 | 23 | New Caledonia | 9 | 86 | FL | 0.912 | [2] |
| *Caranx bucculentus* | 0.023 | 3.033 | 169 | Australia | 5 | 26 | FL | 0.853 | [18] |
| *Caranx ignobilis* | 0.027 | 2.913 | 124 | Hawaii | 21 | 133 | FL | 0.861 | [19] |
| *Caranx lugubris* | 0.020 | 3.001 | 320 | North Marianas | 24 | 76 | FL | 0.840 | [7] |
| *Caranx sexfasciatus* | 0.032 | 2.930 | 24 | Philippines | 5 | 23 | FL | 0.877 | [20] |
| *Caranx tille* | 0.032 | 2.930 | Copied from C. sexfasciatus, same body shape. |  | N/A |  | FL | 0.877 |  |
| *Elagatis bipinnulata* | 0.014 | 2.920 | ?? | Philippines | ?? | ?? | FL | 0.840 | [21] |
| *Seriola dumerili* | 0.023 | 2.847 | 298 | Croatia | 32 | 160 | TL | 1.000 | [22] |
| *Seriola rivoliana* | 0.006 | 3.170 | 88 | Vanuatu | 24 | 88 | FL | 0.903 | [5] |
| *Erythrocles schlegelii* | 0.011 | 3.040 | Based on body shape. |  | N/A |  | FL | 0.906 | [8] |
| *Argyrops spinifer* | 0.055 | 2.670 | 745 | Oman | ?? | ?? | TL | 0.901 | [23] |
| *Dentex carpenteri* | 0.023 | 2.930 | Based on body shape. |  | N/A |  | FL | 0.919 | [8] |
| *Glaucosoma buergeri* | 0.046 | 2.725 | 215 | Australia | 10 | 55 | TL | 1.000 | [24] |
| *Diagramma labiosum* | 0.014 | 2.988 | Copied from C. sexfasciatus, same body shape. |  | N/A |  | FL | 0.901 |  |
| *Diagramma pictum* | 0.014 | 2.988 | 547 | New Caledonia | 7 | 75 | FL | 0.901 | [2] |
| *Pomadasys kaakan* | 0.017 | 2.985 | 101 | Pakistan | 10 | 22 | TL | 1.000 | [25] |
| *Cookeolus japonicus* | 0.047 | 2.700 | 33 | East China Sea | 15 | 47 | TL | 1.000 | [26] |
| *Sphyraena barracuda* | 0.006 | 3.011 | 179 | New Caledonia | 19 | 63 | FL | 0.885 | [2] |
| *Sphyraena forsteri* | 0.005 | 3.034 | 95 | New Caledonia | 9 | 60 | FL | 0.885 | [2] |
| *Sphyraena putnamae* | 0.008 | 2.931 | 226 | New Caledonia | 20 | 104 | FL | 0.906 | [9] |
| *Parascolopsis eriomma* | 0.012 | 2.990 | Based on body shape. |  | N/A |  | FL | 0.935 | [8] |
| *Ostichthys japonicus* | 0.018 | 3.020 | Based on body shape. |  | N/A |  | FL | 0.957 | [8] |
| *Rachycentron canadum* | 0.003 | 3.088 | 288 | USA | 11 | 154 | FL | 0.902 | [27] |
| *Protonibea diacanthus* | 0.026 | 2.646 | 100 | India | 305 | 608 | TL | 1.000 | [28] |
| *Atrobucca brevis* | 0.026 | 2.646 | 100 | India | 305 | 608 | TL | 1.000 | [28] |

**References for S1 Table:**

1. Ralston S V, Williams HA. Depth distributions, growth and mortality of deep slope fishes from the Mariana Archipelago. Vol. NOAA-TM-NM, NOAA Technical Memorandum NMFS. Honolulu; 1988.

2. Kulbicki M, Guillemot N, Amand M. A general approach to length-weight relationships for New Caledonian lagoon fishes. Cybium. 2005;

3. Uchiyama J, Kazama T. Updated Weight-on-Length Relationships for Pelagic Fishes Caught in the Central North Pacific Ocean and Bottomfishes from the Northwestern Hawaiian Islands. Pacific Islands Fish Sci Cent Adm Rep. 2003;

4. Brouard F, Grandperrin R. Les poissons profonds de la pente recifale externe a Vanuatu. Notes et Documents d’Océanographie No 11. 1985.

5. Pakoa K. Vital Statistics of marine fishes in Vanuatu. Naga, ICLARM Q. 1998;21:27–9.

6. Pauly D, Cabanban A, Torres Jr FSB. Fishery biology of 40 trawl-caught teleosts of western Indonesia. In: Pauly D, Martosubroto P, editors. Baseline studies of biodiversity:the fish resource of western Indonesia. 1996. p. 135–216.

7. Ralston S. Length-weight regressions and condition indices of lutjanids and other deep slope fishes from the Mariana Archipelago. Micronesica. 1988;21:189–97.

8. Froese R, Thorson JT, Reyes RB. A Bayesian approach for estimating length-weight relationships in fishes. J Appl Ichthyol. 2014;

9. Letourneur Y, Kulbicki M, Labrosse P. Length-weight relationships of fishes from coral reefs and lagoons of New Caledonia - an update. Naga, ICLARM Q. 1998;

10. Yeh SY, Chen CY, Liu HC. Age determination and growth of red emperor snapper (Lutjanus sebae) in the Arafura Sea off North Australia. Acta Oceanogr Taiwanica. 1986;16:90–102.

11. McPherson GR, Squire L, O’Brien J. Reproduction of Three Dominant Lutjanus Species of the Great Barrier Reef Inter-Reef Fishery. Asian Fish Sci. 1992;5:15–24.

12. Fry GC, Brewer DT, Venables WN. Vulnerability of deepwater demersal fishes to commercial fishing: Evidence from a study around a tropical volcanic seamount in Papua New Guinea. Fish Res. 2006;

13. Matthews CP, Samuel M. Growth, mortality and length-weight parameters for some Kuwaiti fish and shrimp. Fishbyte. 1991;9(2):3.

14. Yanagawa H. Length-weight relationship of Gulf of Thailand fishes. Naga, ICLARM Q. 1994;17(4):48–52.

15. Mees C. Seychelles demersal fishery: an analysis of data relating to four key demersal species. 1992.

16. Kailola PJ, Williams MJ, Stewart PC, Reichelt RE, McNee A, Grieve C. Australian fisheries resources. Canberra: Bureau of Resources Science; 1993. 422 p.

17. Wang XH, Qiu YS, Zhu GP, Du FY, Sun DR, Huang SL. Length-weight relationships of 69 fish species in the Beibu Gulf, northern South China Sea. J Appl Ichthyol. 2011;

18. Willing RS, Pender PJ. Length-weight relationships for 45 species of fish and three invertebrates from Australia’s northern prawn fishery. Darwin: Northern Territory Department of Primary Industries and Fisheries; 1989. 57 p.

19. Seki MP. Carangidae. In: Uchida RN, Uchiyama JH, editors. Fishery Atlas of the Northwestern Hawaiian Islands. NOAA Tech. Rep. NMFS 38; 1986. p. 86–7.

20. Palla HP, Pagliawan HB, Rodriguez EF, Montano BS, Cacho GT, Gonzales BJ, et al. Length-weight relationship of marine fishes from Palawan, Philippines. Palawan Sci. 2018;

21. Kulbicki M, Mou-Tham G, Thollot P, Wantiez L. Length-weight relationships of fish from the lagoon of New Caledonia. Naga, The ICLARM Quarterly. 1993.

22. Koiul, Skaramuca, Kraljevic, Dulcic, Glamuzina. Age, growth and mortality of the Mediterranean amberjack Seriola dumerili (Risso 1810) from the South-Eastern Adriatic Sea. J Appl Ichthyol. 2001;

23. Al Mamry JM, McCarthy ID, Richardson CA, Ben Meriem S. Biology of the kingsoldier bream (Argyrops spinifer, Forsskål 1775; Sparidae), from the Arabian Sea, Oman. J Appl Ichthyol. 2009;

24. Newman SJ. Age, growth, mortality and population characteristics of the pearl perch, Glaucosoma buergeri Richardson 1845, from deeper continental shelf waters off the Pilbara coast of north-western Australia. J Appl Ichthyol. 2002;

25. Hussain SM, Paperno R, Khatoon Z. Length-weight relationships of fishes collected from the Korangi-Phitti Creek area (Indus delta, northern Arabian Sea). J Appl Ichthyol. 2010;

26. Yagi M, Yamada M, Shimoda M, Uchida J, Kinoshita T, Shimizu K, et al. Length-weight relationships of 22 fish species from the East China Sea. J Appl Ichthyol. 2015;

27. Claro R, Garcia-Arteaga JP. Crecimiento. In: Claro R, editor. Ecología de los peces marinos de Cuba. Mexico: Instituto de Oceanologia Academia de Ciencias de Cuba and Centro de Investigaciones de Quintana Roo (CIQRO); 1994. p. 321–402.

28. Manojkumar PP, Acharya P. Morphometry, length-weight relationshop and food and feeding habits of Otolithoides biauritus (Cantor, 1850) of Bombay waters. J Indian Fish Assoc. 1990;20:31–6.
